# Machine learning-based model for predicting metabolic dysfunction-associated steatotic liver disease using non-invasive parameters in young adults

**DOI:** 10.3389/fendo.2025.1701729

**Published:** 2025-12-16

**Authors:** Kyungchul Song, Yu-Jin Kwon, Eunju Lee, Young Hoon Youn, Su Jung Baik, Hye Sun Lee, Hyun Wook Chae

**Affiliations:** 1 Department of Pediatrics, Yonsei University College of Medicine, Gangnam Severance Hospital, Seoul, Republic of Korea; 2 Department of Family Medicine, Yonsei University College of Medicine, Yongin Severance Hospital, Yongin-si, Republic of Korea; 3 Biostatistics Collaboration Unit, Yonsei University College of Medicine, Seoul, Republic of Korea; 4 Department of Healthcare Research Team, Health Promotion Center, Gangnam Severance Hospital, Yonsei University College of Medicine, Seoul, Republic of Korea

**Keywords:** metabolic dysfunction-associated steatotic liver disease, body composition, body mass index, percentage of body fat, young adult

## Abstract

**Background:**

Metabolic dysfunction-associated steatotic liver disease (MASLD) is increasingly being diagnosed in young adults and is associated with long-term hepatic complications. Early detection remains challenging in asymptomatic individuals, highlighting the need for accurate and non-invasive risk assessment tools.

**Methods:**

We developed and validated a machine learning (ML)-based model to predict MASLD in adults aged 20–40 years. A total of 13,047 participants from the Gangnam Severance Hospital were included in the training set, and 1,335 participants from the Yongin Severance Hospital were included in the external validation set. MASLD was defined as hepatic steatosis on ultrasonography with at least one cardiometabolic risk factor. Three models were constructed using stepwise variable addition: Model 1 (age, sex), Model 2 (Model 1 + body mass index [BMI], mean blood pressure), and Model 3 (Model 2 + bioelectrical impedance analysis [BIA] metrics, including percentage of body fat [PBF] and skeletal muscle index [SMI]). Logistic regression (LR), random forest (RF), and extreme gradient boosting (XGB) were also applied.

**Results:**

In internal validation, Model 3 achieved the highest area under the receiver operating characteristic curve (AUROC): 0.90 (LR), 0.91 (RF), and 0.91 (XGB), with accuracies up to 0.81. External validation confirmed a strong performance with AUROCs of 0.89 (LR), 0.88 (RF), and 0.88 (XGB). BMI and PBF were the strongest predictors, whereas a higher SMI was unexpectedly associated with greater MASLD risk.

**Conclusions:**

Our ML-based model using non-invasive parameters accurately predicted MASLD risk in young adults and may facilitate early screening in clinical practice.

## 1 Introduction

Metabolic dysfunction-associated steatotic liver disease (MASLD), formerly known as non-alcoholic fatty liver disease (NAFLD), is the most common chronic liver disease, with an estimated global prevalence of approximately 38% among adults, currently posing a significant public health burden worldwide (1). Recent epidemiological data suggest that the burden of MASLD is rapidly increasing not only in Western countries, but also in Asian populations, including Korea, due to shifts toward Westernized diets, sedentary lifestyles, and rising rates of obesity and type 2 diabetes (2). While traditionally regarded as a condition of middle-aged and older adults, MASLD is now being increasingly diagnosed in young adults (3), with reported prevalence rates ranging from 10% to 30% in individuals under years age of 40 (4, 5).

The early onset of MASLD is particularly concerning, as it may lead to a longer duration of metabolic liver injury and an elevated lifetime risk of adverse outcomes, such as type 2 diabetes, cardiovascular disease, cirrhosis, and mortality (5, 6). However, early identification of MASLD in asymptomatic young individuals remains challenging, as liver enzyme levels may be within normal ranges and imaging is not routinely performed in low-risk populations (7). Although ultrasonography is widely used to detect hepatic steatosis in clinical practice, it is limited by operator dependence and accessibility in large-scale screening settings (7). Liver biopsy, while definitive, is invasive and unsuitable for young, asymptomatic populations (8). Therefore, there is an urgent need for accurate, non-invasive, and easily applicable screening tools that can stratify MASLD risk, particularly in younger age groups.

In recent years, machine learning (ML) techniques have shown considerable promise in enhancing disease prediction by integrating diverse clinical and metabolic variables (9, 10). Applying these methods to routinely collected non-invasive parameters, such as anthropometric indices, blood pressure, and bioelectrical impedance analysis (BIA) metrics, may provide a practical and scalable approach for early risk assessment of MASLD, particularly among young adults. However, evidence on the performance of ML-based models using BIA-derived information remains limited, particularly for Asian populations.

Therefore, in this study, we aimed to develop and validate an ML-based model to predict MASLD using non-invasive clinical and body composition parameters in Korean adults aged 20–40 years. Specifically, we compared the predictive performance of logistic regression (LR), random forest (RF), and extreme gradient boosting (XGB) algorithms in routine health check-up settings.

## 2 Methods

### 2.1 Ethics statements

This study conformed to the ethical guidelines outlined in the 1975 Declaration of Helsinki and was approved by the Institutional Review Board (IRB) of the Yonsei University Gangnam Severance Hospital (IRB number: 3-2024-0221). All participants provided written informed consent prior to data collection.

### 2.2 Study participants

**Figure 1:**
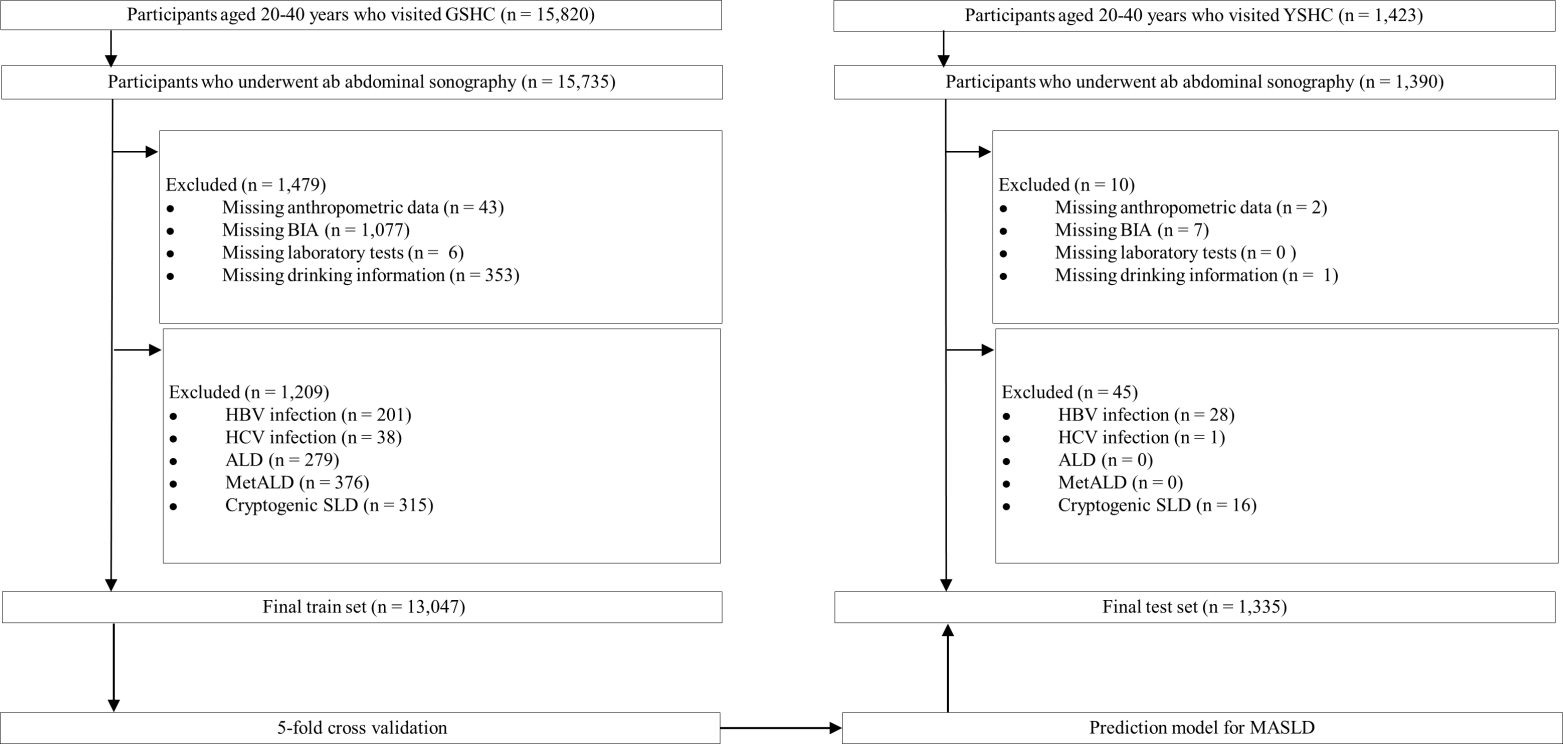
Flowchart of the participant selection. GSHC, Gangnam Severance Hospital Check-up; BIA, bioelectrical impedance analysis; AST, aspartate aminotransferase; ALT, alanine aminotransferase; HBV, hepatitis B virus; HCV, hepatitis C virus; ALD, alcohol-associated/related liver disease; MetALD, metabolic dysfunction and alcohol associated steatotic liver disease; SLD, steatotic liver disease; YSHC, Yongin Severance Hospital Check-up.

In this study, 13,047 young Korean adults aged 20–40 years who participated in the Gangnam Severance Hospital Check-up (GSHC) between January 2017 and December 2023 were included in the training set. A total of 1,335 young adults aged 20–40 years who participated in the Yongin Severance Hospital Check-up (YSHC) from June 2020 to July 2022 were included in the test set. **Figure 1** illustrates the study design and workflow.

### 2.3 Anthropometric measurements and blood pressure

Height was accurately recorded (within 0.1 cm), and body weight was determined using an electronic scale to an accuracy of 0.01 kg. Waist circumference (cm) was measured by a trained nurse at the midpoint between the lower margin of the least palpable rib and top of the iliac crest in the horizontal plane of the waist. Obesity was defined as BMI ≥25 kg/m^2^ and abdominal obesity was defined as waist circumference ≥90 cm for males and ≥85 cm for females according to Asia-Pacific criteria (5) Mean blood pressure (MBP) was calculated as [2×systolic blood pressure + diastolic blood pressure]/3.

### 2.4 Questionnaire

Trained interviewers managed the questionnaire distribution. The survey included questions about coexisting conditions, such as diabetes mellitus, hypertension, dyslipidemia, alcohol consumption, smoking habits, and physical activity levels. Moderate physical activity was defined as engaging in moderate-to-vigorous physical activity for > 150 minutes per week (11).

### 2.5 BIA parameters

Soft lean mass (SLM), percentage of body fat (PBF), total body fat mass (TBF), visceral fat area (VFA), abdominal subcutaneous fat (ASF), and skeletal muscle mass (SMM) were measured using multifrequency bioelectrical impedance analysis (ACCUNIQ BC 720; SELVAS Healthcare, Korea). High PBF was defined as ≥25% for men and ≥35% for women (12). The skeletal muscle index (SMI) was determined by dividing SMM by BMI (13). Low skeletal muscle index (LSMI) was defined as SMI within the lowest quintile for individuals of the same sex aged 20–40 years, based on the sarcopenia criteria established by Janssen et al. (14).

### 2.6 Ultrasonographic analyses

The diagnosis of steatotic liver disease (SLD) was based on abdominal ultrasound results obtained using a 3.5 MHz probe (HDI 5000 at GSHC and EPIQ 7 at YSHC; Philips, Bothell, WA, USA). Ultrasound examinations were performed by one of the three experienced radiologists at each center who were blinded to the participant information. SLD was defined as the presence of at least two of the following ultrasonographic features: (1) a diffuse increase in liver parenchymal echogenicity compared to that in the kidney or spleen, (2) attenuation of the ultrasound beam, and (3) poor visualization of intrahepatic structures (15). Each feature was scored, with 2 indicating a definite presence, 1 indicating a probable presence, and 0 indicating an absence. The total fatty liver score ranged from 0 to 6, with 1–2 indicating mild fat infiltration, 3–4 indicating moderate infiltration, and 5–6 indicating severe infiltration. A score of 0 indicated no hepatic steatosis (15).

### 2.7 Definition of MASLD

MASLD was defined as SLD with one or more cardiometabolic risk factors after excluding individuals with alcoholic liver disease (ALD), metabolic dysfunction, alcohol-associated steatotic liver disease (MetALD), or hepatitis B and C virus infection, based on recent guidelines (8, 16). ALD was defined as hepatic steatosis associated with significant alcohol consumption (> 60 g/day for males and > 50 g/day for females), regardless of metabolic conditions. MetALD was considered as MASLD coexisting with moderate alcohol intake (30–60 g/day in males and 20–50 g/day in females). Cryptogenic SLD was characterized as steatotic liver disease with no clear underlying cause.

### 2.8 Statistical analyses

The baseline characteristics of the participants were compared between groups (normal and MASLD) in the training and test sets, using the independent t-test for continuous variables and the chi-squared test for categorical variables. Continuous variables were presented as mean ± standard deviation, and categorical variables were summarized as counts and percentages.

Multivariable LR analyses were performed to identify the factors associated with MASLD, with the results presented as odds ratios (ORs) and 95% confidence intervals (CIs). Before model fitting, multicollinearity among BMI and fat-related BIA parameters (PBF, TBF, VFA, and ASF) was assessed using the variance inflation factor (VIF). Three models were constructed using non-invasive parameters: Model 1 included age and sex; Model 2 incorporated age, sex, anthropometric measurements, and blood pressure; and Model 3 included BIA parameters. To assess whether the association between PBF and MASLD varied according to BMI, we incorporated an interaction term between BMI and PBF into an additional multivariable logistic regression model. The interaction term (BMI × PBF) was modeled per 10-unit increment of the product term to aid interpretability. Given the statistical significance of the interaction, we further developed BMI-stratified logistic regression models (normal BMI vs. obesity) and evaluated model discrimination using AUROC values. The prediction model was trained using LR, RF, and XGB models from the training set. Internal validation was performed using 5-fold cross validation for hyperparameter optimization, and external validation was performed with the test set (from YSHC). All model hyperparameters used in the final models are provided in Supporting Information **Supplementary Table 1** to ensure reproducibility. Receiver operating characteristic (ROC) curve analyses were conducted to evaluate the discriminative performance of the models. The area under the ROC curve (AUROC) values, sensitivity, specificity, and accuracy were calculated for each model. Pairwise comparisons of the AUROCs between the models were performed using the Delong method. In addition, the AUROC of the LR model was compared with that of the established marker, the hepatic steatosis index (HSI), using the same DeLong test to evaluate relative discriminative performance for MASLD (17). We computed the Youden’s index, positive predictive value (PPV), negative predictive value (NPV), and F1-score to further assess diagnostic performance. Model calibration was evaluated using the calibration intercept, calibration slope, and the Brier score, and was visually assessed using calibration plots. Furthermore, the area under the precision–recall curve (AUPRC) was calculated to better assess discrimination in the presence of class imbalance. Finally, decision curve analysis (DCA) was performed to evaluate the clinical utility and net benefit of each model across a range of threshold probabilities. A scoring system with a nomogram was utilized to predict the probability, and verify the eligibility, of MASLD using the results of the multivariable LR analysis. The contributions of the variables in the models were assessed using Shapely additive explanation (SHAP).

All analyses were conducted using SAS (version 9.4; SAS Inc., Cary, NC, USA) and R (version 4.4.1; R Foundation for Statistical Computing, Vienna, Austria; http://www.R-project.org), with a p-value <0.05 considered statistically significant.

## 3 Results

### 3.1 Baseline characteristics

**Table 1** shows baseline characteristics based on the presence of MASLD in the training and test sets. In the training set, participants with MASLD were older, predominantly male, and had higher BMI, WC, MBP, glucose, total cholesterol, triglycerides, aspartate aminotransferase (AST), alanine aminotransferase (ALT), HSI, SLM, PBF, TBF, VFA, and ASF levels, whereas HDL levels were lower than those in the control group (all p<0.001). Similar trends were observed in the test set, with significant differences in the same variables between MASLD and normal participants.

**Table 1:** Baseline characteristics according to presence of MASLD.

Variables	Train set			Test set		
	Normal (n=9,393)	MASLD (n=3,654)	P-value	Normal (n=975)	MASLD (n=360)	P-value
Age, year	33.81 ± 4.87	35.19 ± 4.24	<0.001	33.10 ± 5.29	34.29 ± 4.69	<0.001
Age group, n (%)			<0.001			0.001
20-25y	648 (6.90)	111 (3.04)		89 (9.13)	17 (4.72)	
26-30y	1826 (19.44)	457 (12.51)		237 (24.31)	64 (17.78)	
31-35y	2762 (29.40)	1075 (29.42)		255 (26.15)	114 (31.67)	
36-40y	4157 (44.26)	2011 (55.04)		394 (40.41)	165 (45.83)	
Sex (Male), n (%)	3310 (35.24)	3059 (83.72)	<0.001	358 (36.72)	284 (78.89)	<0.001
Height, cm	166.80 ± 8.00	173.07 ± 7.53	<0.001	166.58 ± 8.46	172.89 ± 7.62	<0.001
Weight, kg	60.84 ± 11.36	80.47 ± 13.59	<0.001	62.47 ± 11.86	82.09 ± 13.93	<0.001
BMI	21.73 ± 2.79	26.80 ± 3.77	<0.001	22.38 ± 3.06	27.39 ± 3.85	<0.001
Obesity	1147 (12.21)	2379 (65.11)	<0.001	179 (18.36)	258 (71.67)	<0.001
WC, cm	73.48 ± 8.43	89.47 ± 18.76	<0.001	74.15 ± 8.69	88.76 ± 9.18	<0.001
SBP, mmHg	115.16 ± 11.06	121.83 ± 11.56	<0.001	115.24 ± 13.03	124.80 ± 14.15	<0.001
DBP, mmHg	68.65 ± 7.49	71.62 ± 8.44	<0.001	69.60 ± 10.80	76.36 ± 12.39	<0.001
MBP, mmHg	99.66 ± 9.24	105.09 ± 9.93	<0.001	100.03 ± 11.52	108.65 ± 12.81	<0.001
Glucose, mg/dL	92.43 ± 10.17	99.95 ± 15.81	<0.001	91.47 ± 8.94	101.01 ± 20.15	<0.001
TC, mg/dL	197.41 ± 31.95	211.05 ± 36.62	<0.001	184.08 ± 31.11	201.95 ± 38.01	<0.001
TG, mg/dL	91.72 ± 45.14	166.58 ± 116.52	<0.001	87.55 ± 47.78	165.26 ± 124.63	<0.001
HDL, mg/dL	63.15 ± 13.52	49.28 ± 10.62	<0.001	64.14 ± 14.73	48.02 ± 11.06	<0.001
AST, IU/L	24.55 ± 17.79	31.52 ± 18.64	<0.001	22.02 ± 15.12	33.26 ± 24.15	<0.001
ALT, IU/L	19.74 ± 18.48	41.53 ± 32.09	<0.001	18.34 ± 13.62	48.57 ± 43.58	<0.001
HSI	29.28 ± 3.70	37.16 ± 5.86	<.001	30.18 ± 4.17	38.77 ± 6.46	<0.001
SLM, kg	42.36 ± 9.40	53.15 ± 8.48	<0.001	42.73 ± 9.01	53.43 ± 8.62	<0.001
PBF, %	25.47 ± 6.93	28.55 ± 7.21	<0.001	26.39 ± 6.74	29.74 ± 6.80	<0.001
TBF	15.43 ± 5.05	23.30 ± 8.54	<0.001	16.52 ± 5.58	24.71 ± 8.47	<0.001
VFA	1.82 ± 0.91	3.67 ± 2.00	<0.001	1.95 ± 0.97	3.69 ± 1.76	<0.001
ASF	13.61 ± 4.20	19.63 ± 6.60	<0.001	14.56 ± 4.68	21.02 ± 6.77	<0.001
SMM	25.42 ± 5.64	31.89 ± 5.09	<0.001	25.64 ± 5.40	32.05 ± 5.17	<0.001
SMI	116.89 ± 19.94	120.08 ± 18.99	<0.001	114.80 ± 19.54	117.97 ± 17.97	0.007
High PBF, n (%)	1458(15.52)	2320(63.49)	<0.001	179(18.36)	248(68.89)	<0.001
LSMI, n (%)	2022 (21.53)	587 (16.06)	<0.001	271 (27.79)	59 (16.39)	<0.001
DM, n (%)	35(0.37)	58(1.59)	<0.001	6(0.62)	12(3.33)	<0.001
Alcohol consumption, glass/day	1.25 ± 3.06	1.03 ± 1.26	<0.001	0.13 ± 0.19	0.16 ± 0.23	0.018
Smoking, n (%) (n = 14,254)	1195 (12.86)	924 (25.48)	<0.001	149 (15.28)	101 (28.06)	<0.001
Moderate Physical activity, n (%) (n = 10,078)	1694 (26.45)	607 (25.96)	0.648	363 (37.23)	122 (33.89)	0.260

Continuous variables are presented mean ± Standard devation and categorical variables as number (percentages).

*p* value is assessed using independent t–test for continuous variables and Chi–square test for categorical variables.

MASLD, metabolic dysfunction–associated steatotic liver disease; BMI, body mass index; WC, waist circumference; SBP, systolic blood pressure, DBP, diastolic blood pressure; MBP, mean blood pressure; TC, total cholesterol; TG, triglycerides; HDL, high-density lipoprotein cholesterol; LDL, low-density lipoprotein cholesterol; AST, aspartate aminotransferase; ALT, alanine aminotransferase; GGT, Gamma-glutamyl transferase’ FLI, fatty liver index; HSI, hepatic steatosis index; SLM, soft lean mass; PBF, percentage of body fat; TBF, total body fat mass; SMM, skeletal muscle mass; VFA, visceral fat area; ASF, abdominal subcutaneous fat; SMI, skeletal muscle mass index; LSMI, low skeletal muscle index.

**Supplementary Table 2** presents the baseline characteristics of the training and test sets. Participants in the test set had a lower proportion of obesity and higher BMI, weight, MBP, DBP, LDL, HSI, PBF, TBF, ASF, and LSMI than did those in the training set. Additionally, alcohol consumption was lower, and the prevalence of moderate physical activity was higher, in the test set.

### 3.2 LR analyses for MASLD

Multicollinearity among BMI and fat-related BIA parameters (PBF, TBF, VFA, and ASF) was assessed using the VIF. TBF, VFA, and ASF showed high multicollinearity with BMI; therefore, only PBF was included as the fat-related parameter in the final model (**Supplementary Table 3**).

Multivariable LR analyses identified significant factors associated with MASLD (**Table 2**). In Model 1, age and male sex were significantly and positively associated with MASLD. In Model 2, BMI and MBP were added to Model 1, and age, male sex, BMI, and MBP were positively associated with MASLD. Model 3 incorporated PBF and SMI, as BIA parameters, and these factors were significantly associated with MASLD. Age, male sex, BMI, MBP, PBF, and SMI were positively associated with MASLD.

**Table 2:** Multivariable logistic regression analyses for MASLD.

Variables	Model1		Model2		Model3	
	OR (95% CI)	P-value	OR (95% CI)	P-value	OR (95% CI)	P-value
Age	1.07 (1.06-1.09)	<0.001	1.08 (1.07-1.09)	<0.001	1.09 (1.07-1.10)	<0.001
Male sex	9.62(8.72-10.61)	<0.001	3.38(3.00-3.80)	<0.001	8.73(7.07-10.79)	<0.001
BMI			1.57 (1.54-1.61)	<0.001	1.38 (1.34-1.41)	<0.001
MBP			1.02 (1.02-1.03)	<0.001	1.02 (1.01-1.03)	<0.001
PBF					1.12 (1.10-1.14)	<0.001
SMI					1.01 (1.00-1.02)	0.003

Model 1: age and sex

Model 2: age, sex, anthropometric measurement, and BP

Model 3: age, sex, anthropometric measurement, BP, and BIA

MASLD, metabolic dysfunction–associated steatotic liver disease; OR, odds ratio; CI, confidence interval; BMI, body mass index; MBP, mean blood pressure; PBF, percentage of body fat; SMI, skeletal muscle mass index; BP, blood pressure; BIA, bioelectric impedance analysis.

To further examine whether the association between PBF and MASLD differed according to BMI level, we incorporated an interaction term between BMI and PBF into an additional logistic regression model. The interaction term was statistically significant (p = 0.036), indicating that the effect of PBF on MASLD risk varied across BMI strata. In BMI-stratified analyses, PBF showed a stronger association with MASLD in the normal BMI group (OR 1.23, 95% CI 1.20–1.26), whereas the association was attenuated in the obesity group (OR 1.17, 95% CI 1.14–1.20) (data not shown).

### 3.3 ROC analyses of each model for predicting MASLD

The ROC analyses revealed progressive improvements in the AUROC values across the models (**Table 3**). In both internal and external validation, model performance improved with the sequential addition of variables. Model 1, which included only age and sex, demonstrated moderate discriminative ability. The inclusion of BMI and MBP in Model 2 resulted in a increase in predictive accuracy across all machine learning algorithms. Model 3, which additionally incorporated PBF and SMI, yielded the highest performance.

**Table 3:** ROC analyses for MASLD.

Models			AUROC (95% CI)	p-value	Sensitivity	Specificity	Youden’s Index	Accuracy	PPV	NPV	F1-Score	Calibration intercept	Calibration slope	Brier Score	AUPRC
Internal validation	Model 1	LR	0.78 (0.77-0.79)	<0.001	0.82 (0.80-0.83)	0.68 (0.67-0.69)	0.49 (0.49-0.49)	0.71 (0.71-0.72)	0.49 (0.48-0.51)	0.90 (0.90-0.91)	0.62 (0.60-0.63)	0.000	1.000	0.160	0.519
		RF	0.75 (0.74-0.76)	<0.001	0.75 (0.74-0.77)	0.72 (0.71-0.73)	0.47 (0.47-0.47)	0.73 (0.72-0.74)	0.51 (0.50-0.52)	0.88 (0.88-0.89)	0.61 (0.59-0.62)	0.062	0.155	0.170	0.506
		XGB	0.78 (0.77-0.79)	<0.001	0.82 (0.81-0.83)	0.67 (0.66-0.68)	0.49 (0.49-0.49)	0.71 (0.70-0.72)	0.49 (0.48-0.50)	0.91 (0.90-0.91)	0.62 (0.60-0.63)	0.009	1.014	0.159	0.520
	Model 2	LR	0.90 (0.89-0.90)	<0.001	0.86 (0.85-0.87)	0.78 (0.77-0.79)	0.63 (0.63-0.63)	0.80 (0.79-0.81)	0.60 (0.59-0.61)	0.93 (0.93-0.94)	0.71 (0.69-0.72)	0.000	1.000	0.119	0.763
		RF	0.87 (0.87-0.88)	<0.001	0.87 (0.86-0.88)	0.78 (0.77-0.79)	0.65 (0.65-0.65)	0.80 (0.80-0.81)	0.61 (0.59-0.62)	0.94 (0.93-0.94)	0.71 (0.70-0.73)	-0.066	0.215	0.128	0.742
		XGB	0.91 (0.90-0.91)	<0.001	0.90 (0.89-0.91)	0.76 (0.75-0.77)	0.65 (0.65-0.65)	0.80 (0.79-0.80)	0.59 (0.58-0.60)	0.95 (0.94-0.95)	0.71 (0.70-0.73)	0.078	1.472	0.113	0.784
	Model 3	LR	0.90 (0.90-0.91)	<0.001	0.86 (0.85-0.87)	0.79 (0.78-0.80)	0.65 (0.65-0.65)	0.81 (0.80-0.81)	0.61 (0.60-0.62)	0.93 (0.93-0.94)	0.71 (0.70-0.73)	0.000	1.000	0.116	0.773
		RF	0.91 (0.90-0.91)	<0.001	0.90 (0.89-0.91)	0.76 (0.75-0.77)	0.66 (0.66-0.66)	0.80 (0.79-0.81)	0.59 (0.58-0.61)	0.95 (0.95-0.96)	0.72 (0.70-0.73)	0.010	0.391	0.119	0.783
		XGB	0.91 (0.91-0.92)	<0.001	0.89 (0.88-0.90)	0.78 (0.77-0.79)	0.67 (0.67-0.67)	0.81 (0.81-0.82)	0.61 (0.60-0.63)	0.95 (0.94-0.95)	0.73 (0.71-0.74)	0.042	1.331	0.108	0.801
External validation	Model 1	LR	0.74 (0.71-0.77)	<0.001	0.75 (0.70-0.79)	0.66 (0.63-0.69)	0.41 (0.41-0.41)	0.69 (0.66-0.71)	0.45 (0.41-0.49)	0.88 (0.85-0.90)	0.56 (0.52-0.61)	-0.126	0.820	0.169	0.448
		RF	0.69 (0.67-0.72)	<0.001	0.66 (0.61-0.70)	0.73 (0.71-0.76)	0.39 (0.39-0.39)	0.71 (0.69-0.74)	0.48 (0.43-0.52)	0.85 (0.83-0.88)	0.55 (0.50-0.60)	-0.094	0.133	0.178	0.416
		XGB	0.74 (0.71-0.77)	<0.001	0.76 (0.71-0.80)	0.66 (0.63-0.69)	0.42 (0.41-0.42)	0.68 (0.66-0.71)	0.45 (0.41-0.49)	0.88 (0.86-0.90)	0.56 (0.52-0.61)	-0.117	0.830	0.169	0.434
	Model 2	LR	0.88 (0.86-0.90)	<0.001	0.88 (0.85-0.91)	0.73 (0.70-0.76)	0.61 (0.61-0.61)	0.77 (0.75-0.79)	0.54 (0.50-0.59)	0.94 (0.93-0.96)	0.67 (0.63-0.72)	-0.393	0.866	0.124	0.719
		RF	0.85 (0.83-0.88)	<0.001	0.88 (0.85-0.92)	0.69 (0.66-0.72)	0.58 (0.57-0.58)	0.74 (0.72-0.77)	0.51 (0.48-0.55)	0.94 (0.92-0.96)	0.65 (0.61-0.69)	-0.423	0.264	0.134	0.667
		XGB	0.88 (0.86-0.90)	<0.001	0.90 (0.87-0.93)	0.70 (0.67-0.73)	0.60 (0.60-0.60)	0.75 (0.73-0.78)	0.53 (0.49-0.56)	0.95 (0.94-0.97)	0.66 (0.62-0.71)	-0.324	1.282	0.123	0.715
	Model 3	LR	0.89 (0.87-0.90)	<0.001	0.88 (0.85-0.91)	0.72 (0.70-0.75)	0.60 (0.60-0.61)	0.77 (0.74-0.79)	0.54 (0.50-0.58)	0.94 (0.93-0.96)	0.67 (0.63-0.71)	-0.386	0.881	0.120	0.729
		RF	0.88 (0.86-0.90)	<0.001	0.92 (0.89-0.95)	0.66 (0.63-0.69)	0.58 (0.58-0.59)	0.73 (0.71-0.76)	0.50 (0.46-0.54)	0.96 (0.94-0.97)	0.65 (0.61-0.69)	-0.403	0.397	0.127	0.708
		XGB	0.88 (0.87-0.90)	<0.001	0.89 (0.86-0.92)	0.71 (0.68-0.74)	0.60 (0.60-0.60)	0.76 (0.74-0.78)	0.53 (0.49-0.57)	0.95 (0.93-0.96)	0.67 (0.62-0.71)	-0.387	1.129	0.120	0.727

ROC, receiver operating characteristic curve; MASLD, metabolic dysfunction–associated steatotic liver disease; AUROC, area under the receiver operating characteristic curve; PPV, positive predictive value; NPV, negative predictive value; AUPRC, area under the precision-recall curve; LR, logistic regression analysis; RF, random forest; XGB, extreme gradient boosting.

**Figure 2:**
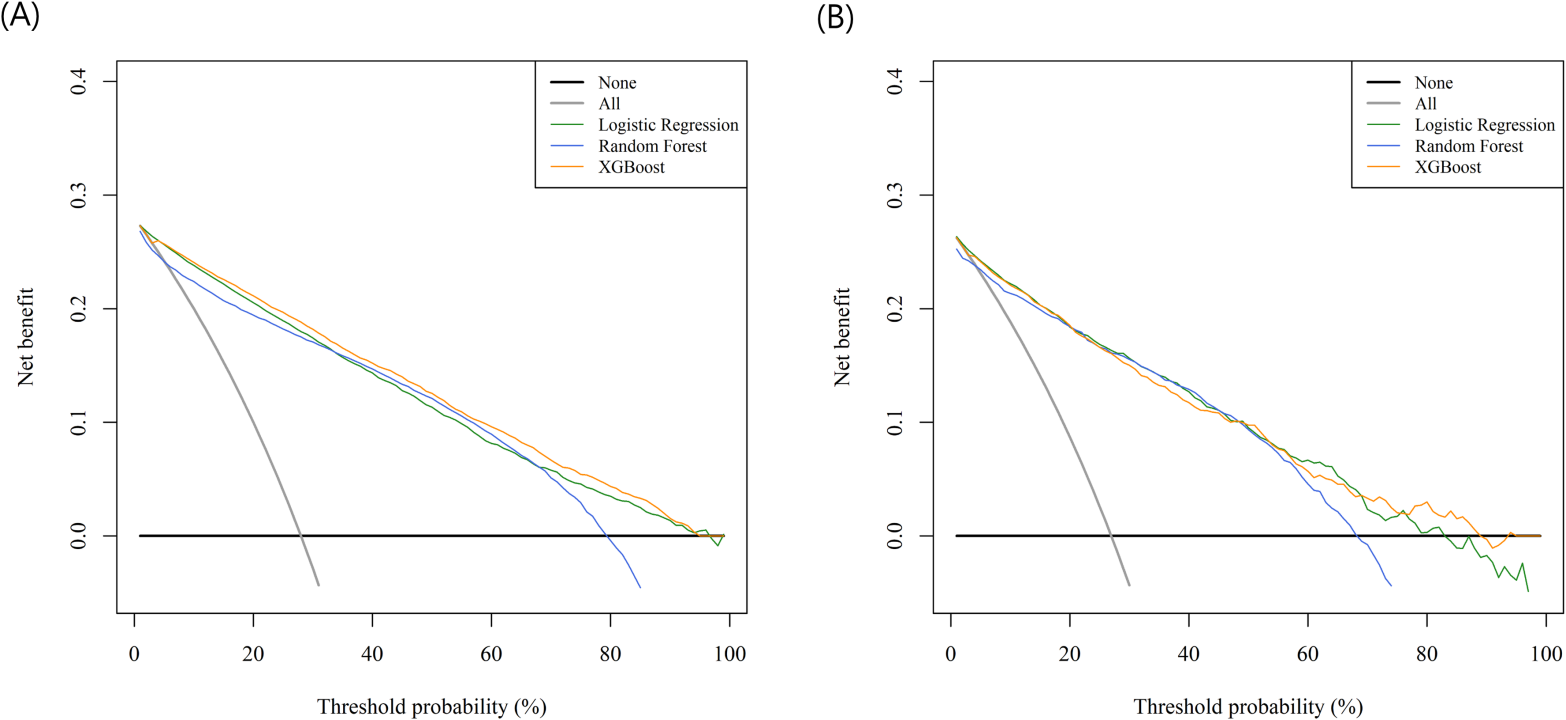
Decision curve analysis for Model 3 in internal and external validation datasets. **(A)** Decision curve analysis of Model 3 in the internal validation dataset. **(B)** Decision curve analysis of Model 3 in the external validation dataset. XGB, extreme gradient boosting.

In internal validation, Model 3 achieved AUROCs of 0.90 for LR, 0.91 for RF, and 0.91 for XGB, with corresponding AUPRCs of 0.77–0.80 and low Brier scores (0.108–0.119), with corresponding AUPRCs of 0.77–0.80 and low Brier scores (0.108–0.119), indicating good agreement between predicted and observed outcomes. The calibration intercepts were close to 0 and slopes near 1 across models, suggesting well-calibrated predictions. PPV, NPV, and F1 scores also increased progressively, with the highest values observed in Model 3. The calibration plots showed that the predicted probabilities from LR and XGBoost were closely aligned with the ideal 45° reference line, whereas those from the random forest model demonstrated a slightly larger deviation (**Supplementary Figures 1A-C**). DCA further demonstrated that all three algorithms in Model 3 provided greater net benefit than the treat-all or treat-none strategies across clinically relevant threshold probabilities (**Figure 2A**).

In external validation, the corresponding AUROCs were 0.89, 0.88, and 0.88, with AUPRCs of 0.71–0.73 and low Brier scores (0.120–0.127), indicating stable predictive performance (**Table 3**). The calibration intercepts ranging from –0.42 to –0.32 and slopes near 1, demonstrating good overall agreement. The calibration plots showed that the predicted probabilities from LR and XGBoost were closely aligned with the ideal 45° reference line, whereas those from the random forest model demonstrated a slightly larger deviation. Calibration plots indicated closer alignment with the ideal 45° reference line for LR and XGBoost than for random forest (**Supplementary Figures 1D-F**). DCA for Model 3 showed that all algorithms provided greater net benefit than the treat-all or treat-none strategies across clinically relevant threshold probabilities (**Figure 2B**).

In the pairwise comparison, Model 2 was superior to Model 1, whereas Model 3 demonstrated superior predictive performance to both Models 1 and 2 across all comparisons (**Supplementary Table 4**). In addition, in the internal validation, the AUROC of the LR model (0.901) was significantly higher than that of the hepatic steatosis index (HSI; 0.893, p < 0.001). In the external validation, the AUROCs of the two models (0.886 vs. 0.889) were comparable, with no statistically significant difference (p = 0.631).

Consistent with these association findings, discriminatory performance of PBF also differed by BMI category (**Supplementary Figure 2**). In the internal validation dataset, AUROC values were 0.85 for the normal BMI group and 0.73 for the obesity group. A similar pattern was observed in the external validation dataset, with AUROCs of 0.83 and 0.72, respectively. These results indicate that PBF provides greater incremental predictive value for MASLD among individuals with normal BMI.

### 3.4 Scoring system with nomogram

A scoring system based on a multivariate LR model was developed to calculate the probability of developing MASLD (**SI Figure 3**). The probability is determined using the following formula:

Probability (MASLD) = 1/(1 + exp(−y)).

where y is computed as:

y = −14.743 + 0.083×Age − 2.167×Sex + 0.319×BMI + 0.020×MBP + 0.111×PBF + 0.010×SMI.

**Figure 3:**
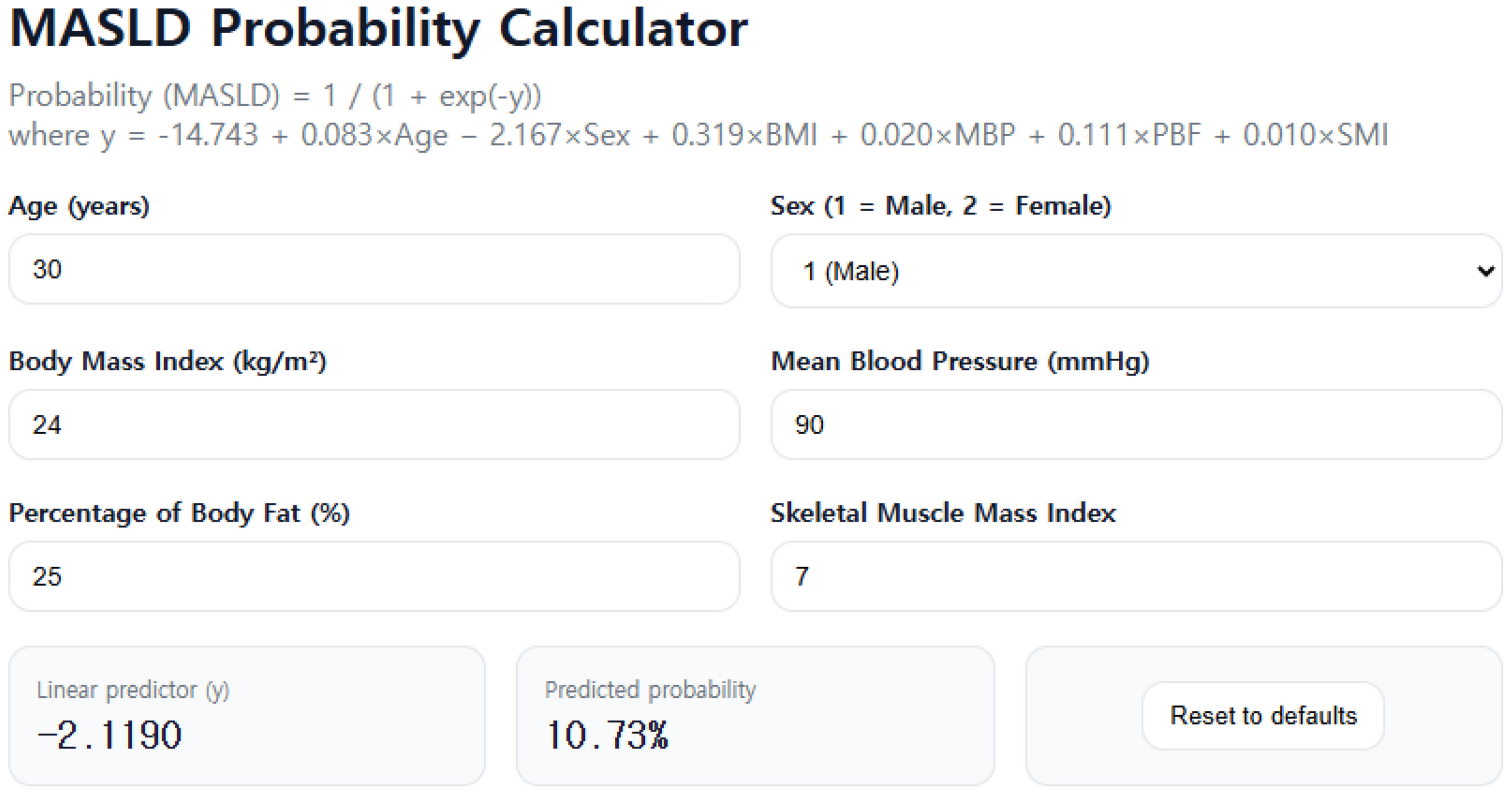
Example of MASLD probability estimation using the web-based MASLD probability calculator. MASLD, metabolic dysfunction-associated steatotic liver disease.

To enhance clinical applicability, we also developed an interactive web-based MASLD probability calculator that computes disease probability using these coefficients. The source code has been made publicly available on GitHub (https://github.com/endosong/MASLD-Probability-Calculator.git). An example of probability estimation using this calculator is presented in **Figure 3**.

### 3.5 Contribution of the variables

**Figure 4:**
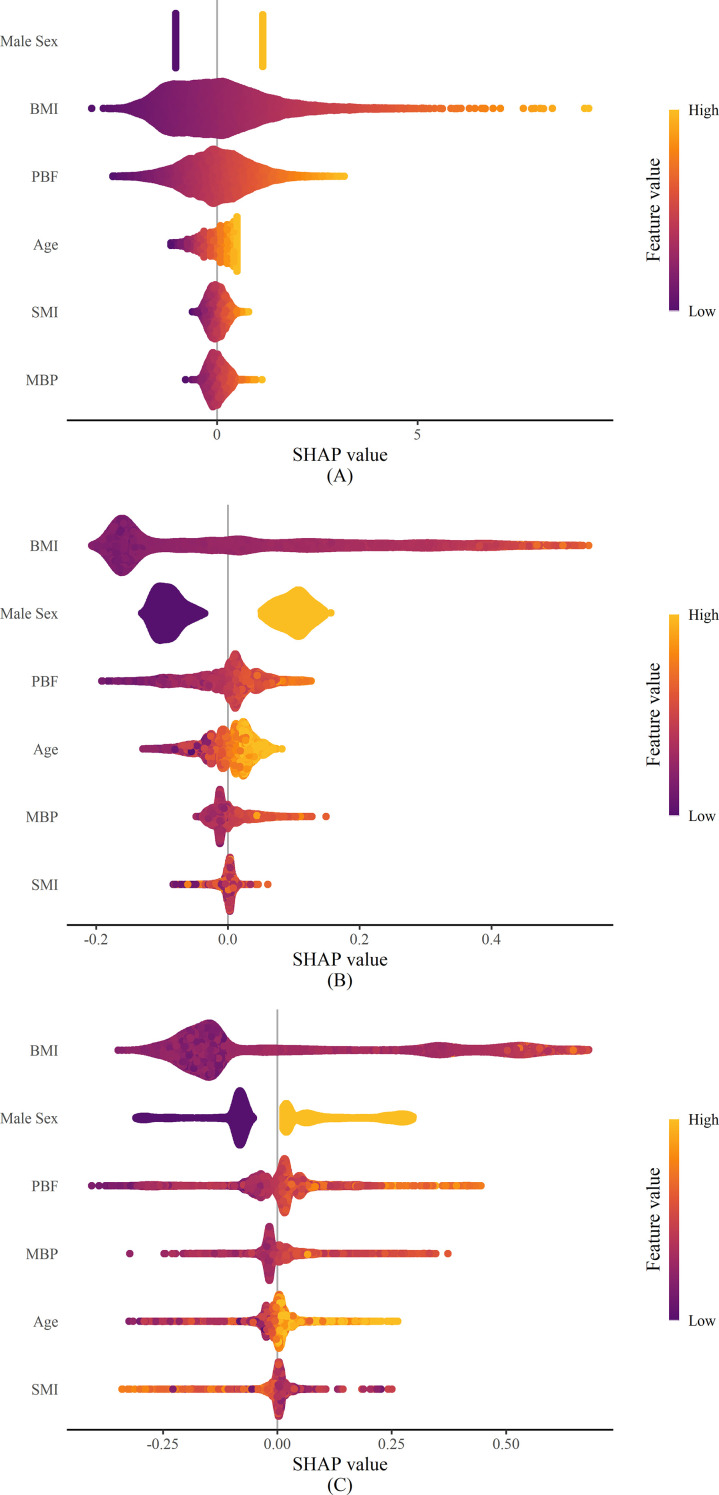
SHAP summary plot for contribution of the variables for predicting MASLD using the training set.**(A)** SHAP summary plot of the prediction model using logistic regression analysis using the training set **(B)** SHAP summary plot of the prediction model using random forest using the training set **(C)** SHAP summary plot of the prediction model using XGB using the training setThe color of the plot indicates whether a parameter has a relatively high or low value within the participant dataset. The horizontal position represents the degree of influence of the parameter on the prediction, with the placement reflecting a stronger or weaker impact.SHAP, Shapley additive explanation; MASLD, metabolic dysfunction-associated steatotic liver disease; BMI, body mass index; PBF, percentage of body fat; MBP, mean blood pressure; SMI, skeletal muscle mass index; XGB, extreme gradient boosting.

**Figure 4** presents SHAP summary plots demonstrating the contribution of each variable to MASLD prediction across logistic regression (4A), random forest (4B), and XGB (4C) models in the external validation dataset. In all three models, male sex and BMI were identified as the parameters with the highest contributions to the prediction, followed by PBF. In the LR model, the next most influential parameters were age, SMI, and MBP, in that order, while the order shifted to age, MBP, and SMI for RF, and MBP, age, and SMI for XGB.

**Supplementary Figure 4** presents the SHAP values of the LR model in the external validation dataset stratified by age group. In all age groups, sex and BMI showed the highest contributions to MASLD prediction. The relative importance of age, PBF, SMI, and MBP varied across age groups.

**Supplementary Figure 5** shows the SHAP values of the LR model in the external validation dataset stratified by BMI category. In the normal BMI group, sex showed the highest contribution to MASLD prediction, followed by BMI, PBF, age, SMI, and MBP. In the obesity group, BMI had the greatest contribution, followed by sex, PBF, age, MBP, and SMI.

## 4 Discussion

In this study, we developed and validated ML-based models to predict MASLD in young adults using simple, non-invasive parameters derived from routine health check-ups. Model performance progressively improved with the sequential addition of variables, and Model 3, which incorporated BIA-derived measures, showed the best overall performance with high AUROC and AUPRC values and good calibration. In the comparison among algorithms within Model 3, LR, random forest, and XGBoost demonstrated comparable discriminative performance and clinical utility in both internal and external validation, as shown by ROC and decision curve analyses, indicating stable and consistent predictive performance across datasets. Moreover, our model was not inferior to the HSI, a conventional MASLD marker that requires blood testing, despite relying solely on non-invasive parameters. Our findings are consistent with those of previous studies, showing that obesity and visceral adiposity are key contributors to MASLD pathogenesis in younger populations (5, 18). Importantly, our model incorporated routinely collected non-invasive variables, making it highly applicable to primary care and health check-up settings where advanced imaging is not routinely available.

Several ML-based models have been developed for predicting MASLD using accessible clinical and demographic data (19–21). A recent study from China developed machine learning models to predict fatty liver disease using physical and biochemical variables in a general adult population with internal validation (22). Another large-scale Chinese study (n=10,007) using eight basic variables (e.g., age, waist/hip circumference, comorbidities) developed ML models with strong performance, with AUROCs of 0.798–0.806 in internal testing and 0.831 in external validation; multilayer perceptron and XGBoost also performed well with AUROCs of 0.823 and 0.784, respectively (19). Another recent model built on key metabolic indices, such as waist circumference, homeostatic model assessment of insulin resistance, triglycerides, and glucose, achieved a mean AUROC of 0.960, underscoring the relevance of insulin resistance-related features (20). In addition, a European study demonstrated that advanced MASLD outcomes, including metabolic dysfunction-associated steatohepatitis and fibrosis, could be predicted with high accuracy (AUROC 0.719–0.994) by using 19 routine clinical indicators (21). However, these models were primarily developed for middle-aged Western or Chinese populations and did not incorporate direct body composition data. Furthermore, conventional models and markers such as the HSI require blood tests, which limit their applicability in large-scale or community-based screening settings. In contrast, our study focused on a young Asian cohort and developed a model using only simple, non-invasive variables readily available in routine health check-up settings, namely age, sex, blood pressure, and body composition measures from BIA and validated the model with the independent test set. This approach highlights the potential for early MASLD risk assessment using easily obtainable data, even in non-hospital or community settings, without the need for laboratory or imaging tests. Despite relying on these basic parameters, our model demonstrated a high predictive performance. This highlights its practicality and strong potential for real-world applications, particularly in primary care and large-scale screening programs, where simplicity, cost efficiency, and accessibility are critical.

Importantly, our findings demonstrate that incorporating BIA-derived parameters significantly improves model performance. In our study, the model performance improved with each stepwise addition of variables. Model 1 showed moderate accuracy (AUROC up to 0.74), whereas Model 2, which included anthropometric and blood pressure data, improved significantly (AUROC up to 0.88) in the external validation. Model 3, which added BIA-derived parameters, achieved the highest performance, with AUROCs of 0.90–0.91 (internal validation) and 0.88–0.89 (external validation)—significantly outperforming Model 2, highlighting the value of body composition data in enhancing MASLD prediction.

Among these parameters, BMI and PBF emerged as the most influential contributors to MASLD prediction in all models, as confirmed by SHAP analysis. This finding is biologically plausible and consistent with existing pathophysiological knowledge. BMI reflects overall adiposity, which is closely linked to insulin resistance, hepatic fat accumulation, and systemic inflammation, which are the hallmarks of MASLD pathogenesis (23). However, BMI alone cannot distinguish between fat and lean mass (24). Therefore, the inclusion of PBF offers complementary information by specifically quantifying the proportion of body fat directly related to ectopic fat deposition and lipotoxicity in the liver (25, 26). Adipose tissue, particularly when in excess, secretes pro-inflammatory adipokines (e.g., TNF-α, IL-6) and reduces adiponectin levels, contributing to systemic insulin resistance and hepatic steatosis (27). Thus, the combined use of BMI and PBF provides a more nuanced assessment of metabolic risk than either metric alone, allowing for better identification of young individuals at risk of MASLD despite potentially normal body weight.

Our study found that a higher SMI was independently associated with an increased risk of MASLD, which contrasts with the conventional view that greater muscle mass is metabolically protective (28). Several factors could explain this unexpected finding. First, SMI derived from BIA may overestimate muscle mass in individuals with increased visceral fat or fluid retention, potentially confounding its association with metabolic risk (29, 30). Second, emerging evidence suggests that muscle quantity alone does not adequately capture metabolic health (31). The metabolic function of skeletal muscles is strongly influenced by muscle quality, including fiber type composition and fat infiltration (myosteatosis) (32). Individuals with obesity often exhibit a predominance of type II (fast-twitch), glycolytic, and insulin-resistant fibers, which may contribute to systemic insulin resistance, despite having greater muscle mass (33). Moreover, myosteatosis, characterized by ectopic fat accumulation within the muscle tissue, has been associated with hepatic steatosis, systemic inflammation, and metabolic dysfunction, independent of total muscle quantity (34). Consistent with our findings, a recent meta-analysis reported that sarcopenia significantly increased the risk of MASLD, whereas SMI alone was not associated with MASLD (28), further supporting the notion that muscle mass is not a sufficient marker of metabolic health. These findings underscore the limitations of relying solely on muscle mass and highlight the importance of incorporating measures of muscle strength or function, as these parameters may better reflect the true muscle quality and metabolic resilience (35).

Despite these strengths, several limitations of this study must be acknowledged. The study population was limited to Korean adults undergoing health checkups, which may limit generalizability to other ethnic or clinical populations. Additionally, the cross-sectional nature of the data precluded causal inferences. Although BIA may be less accurate than gold-standard methods, such as dual-energy X-ray absorptiometry or magnetic resonance imaging, it offers several practical advantages. BIA is non-invasive, rapid, cost-effective, and easily applicable in routine clinical and screening settings, making it particularly suitable for large-scale population studies (36).

## 5 Conclusion

In conclusion, we developed and validated an ML-based model that accurately predicts MASLD in young Korean adults using simple, non-invasive parameters routinely collected during health checkups, including basic clinical measures and BIA-derived body composition data. This model demonstrates that MASLD risk can be effectively estimated using easily accessible variables such as BMI, blood pressure, and BIA indices, even in non-hospital settings, thereby facilitating early detection and preventive management in the community. Importantly, our findings also highlight the added clinical value of incorporating PBF, particularly for identifying high-risk individuals with normal BMI who may otherwise be overlooked. This underscores the practical utility and scalability of the model for early MASLD risk assessment in real-world primary care and large-scale screening settings. Further validation in diverse populations and longitudinal studies are warranted to confirm the models broad applicability and long-term prognostic value.

## Data Availability

The raw data supporting the conclusions of this article will be made available by the authors, without undue reservation.
