# Supplementary material for: Machine learning-based model for predicting metabolic dysfunction-associated steatotic liver disease using non-invasive parameters in young adults
Source: Front Endocrinol (Lausanne). 2025 Dec 16;16:1701729. doi: 10.3389/fendo.2025.1701729 (PMC12747903; doi:10.3389/fendo.2025.1701729)
Supplement: Supplementary file 1 [file DataSheet1.docx]

Supporting information table 1. Hyperparameters used for each machine learning algorithm.

| Random Forest | | | | XGBoost | | | |
| --- | --- | --- | --- | --- | --- | --- | --- |
| R package : {randomForest} | | | | R package : {xgboost} | | | |
| Parameter | Model1 | Model2 | Model3 | Parameter | Model1 | Model2 | Model3 |
| Number of tree | 100 | 100 | 300 | Number of boosting | 100 | 50 | 300 |
| Number of features per split | 1 | 3 | 2 | Maximum depth | 5 | 5 | 5 |
| Minimum samples per leaf | 3 | 3 | 7 | Learning Rate | 0.1 | 0.05 | 0.01 |
| Maximum number of leaves | 30 | 30 | 50 | Gamma | 0 | 0 | 1 |
|  | | | | Minimum child weight | 2 | 2 | 2 |
|  |  |  |  | Column sample by tree | 0.7 | 0.8 | 0.9 |
|  |  |  |  | Subsample ratio | 0.7 | 0.7 | 0.7 |

Supporting information table 2. Baseline characteristics of the training set and test set

|  | Total | Train set (n=13,047) | Test set (n=1,335) | p-value |
| --- | --- | --- | --- | --- |
| Age, year | 34.12±4.79 | 34.19±4.74 | 33.42±5.16 | <0.001 |
| Age group, n (%) |  |  |  | <0.001 |
| 20-25y | 865(6.01) | 759(5.82) | 106(7.94) |  |
| 26-30y | 2584(17.97) | 2283(17.50) | 301(22.55) |  |
| 31-35y | 4206(29.24) | 3837(29.41) | 369(27.64) |  |
| 36-40y | 6727(46.77) | 6168(47.28) | 559(41.87) |  |
| Sex (Male), n (%) | 7011 (48.75) | 6369 (48.82) | 642 (48.09) | 0.613 |
| Height, cm | 168.53±8.39 | 168.55±8.36 | 168.28±8.70 | 0.273 |
| Weight, kg | 66.47±14.94 | 66.34±14.91 | 67.76±15.19 | <0.001 |
| BMI | 23.20±3.86 | 23.15±3.84 | 23.73±3.97 | <0.001 |
| Obesity | 3963 (27.56) | 3526 (27.03) | 437 (32.73) | <0.001 |
| WC, cm | 77.97±13.92 | 77.96±14.19 | 78.09±10.95 | 0.684 |
| SBP, mmHg | 117.10±11.84 | 117.03±11.59 | 117.82±13.99 | 0.046 |
| DBP, mmHg | 69.66±8.32 | 69.48±7.88 | 71.42±11.64 | <0.001 |
| MBP, mmHg | 101.29±10.04 | 101.18±9.75 | 102.36±12.48 | <0.001 |
| Glucose, mg/dL | 94.49±12.59 | 94.54±12.48 | 94.05±13.62 | 0.204 |
| Uric acid, mg/dL | 5.47±1.51 | 5.49±1.52 | 5.24±1.48 | <0.001 |
| TC, mg/dL | 200.08±34.08 | 201.23±33.88 | 188.90±34.03 | <0.001 |
| TG, mg/dL | 112.30±80.37 | 112.68±79.99 | 108.51±83.89 | 0.082 |
| HDL, mg/dL | 59.31±14.34 | 59.27±14.21 | 59.79±15.57 | 0.234 |
| LDL, mg/dL | 119.45±29.68 | 119.00±29.23 | 123.81±33.45 | <0.001 |
| AST, IU/L | 26.37±18.34 | 26.50±18.30 | 25.05±18.67 | 0.006 |
| ALT, IU/L | 25.90±25.46 | 25.84±25.10 | 26.49±28.76 | 0.428 |
| HSI | 31.58±5.72 | 31.49±5.65 | 32.50±6.20 | <0.001 |
| SLM, kg | 45.41±10.33 | 45.38±10.36 | 45.62±10.09 | 0.428 |
| PBF, % | 26.42±7.13 | 26.33±7.15 | 27.29±6.91 | <0.001 |
| TBF | 17.74±7.19 | 17.64±7.16 | 18.73±7.43 | <0.001 |
| VFA | 2.34±1.54 | 2.34±1.55 | 2.42±1.46 | 0.054 |
| ASF | 15.39±5.72 | 15.30±5.68 | 16.30±6.05 | <0.001 |
| SMM | 27.24±6.20 | 27.23±6.21 | 27.37±6.05 | 0.429 |
| SMI | 117.58±19.69 | 117.78±19.73 | 115.65±19.17 | <0.001 |
| High PBF, n (%) | 4205(29.24) | 3778(28.96) | 427(31.99) | 0.021 |
| LSMI | 2939 (20.44) | 2609 (20.00) | 330 (24.72) | <0.001 |
| DM, n (%) | 111(0.77) | 93(0.71) | 18(1.35) | 0.011 |
| Alcohol consumption, glass/day | 1.09±2.57 | 1.19±2.68 | 0.14±0.20 | <0.001 |
| Smoking, n (%) (n = 14,254) | 2369 (16.62) | 2119 (16.40) | 250 (18.73) | 0.030 |
| Moderate Physical activity, n (%)  (n = 10,078) | 2786 (27.64) | 2301 (26.32) | 485 (36.33) | <0.001 |
| MASLD, n (%) | 4014 (27.91) | 3654 (28.01) | 360 (26.97) | 0.420 |

Continuous variables are presented mean±Standard devation and categorical variables as number (percentages).

*p* value is assessed using independent t–test for continuous variables and Chi–square test for categorical variables.

MASLD, metabolic dysfunction–associated steatotic liver disease; BMI, body mass index; WC, waist circumference; SBP, systolic blood pressure, DBP, diastolic blood pressure; MBP, mean blood pressure; TC, total cholesterol; TG, triglycerides; HDL, high-density lipoprotein cholesterol; LDL, low-density lipoprotein cholesterol; AST, aspartate aminotransferase; ALT, alanine aminotransferase; GGT, Gamma-glutamyl transferase’ FLI, fatty liver index; HSI, hepatic steatosis index; SLM, soft lean mass; PBF, percentage body fat; TBF, total body fat mass; SMM, skeletal muscle mass; VFA, visceral fat area; ASF, abdominal subcutaneous fat; SMI, skeletal muscle mass index; LSMI, low skeletal muscle index.

Supporting information table 3. Multicollinearity of BMI, PBF, TBF, VFA, and ASF

|  | VIF |
| --- | --- |
| BMI | 8.192 |
| PBF | 8.319 |
| TBF | 66,919,519 |
| VFA | 3,128,582 |
| ASF | 42,090,799 |

BMI, body mass index; PBF, percentage body fat; TBF, total body fat mass; SMM, skeletal muscle mass; VFA, visceral fat area; ASF, abdominal subcutaneous fat

Supporting information table 4. Pairwise comparison of AUCs between the models

|  |  |  | Model 1 | | | Model 2 | | | Model 3 | | |
| --- | --- | --- | --- | --- | --- | --- | --- | --- | --- | --- | --- |
|  |  |  | LR | RF | XGB | LR | RF | XGB | LR | RF | XGB |
| Internal validation | Model 1 | LR | ref |  |  |  |  |  |  |  |  |
|  |  | RF | <0.001 | ref |  |  |  |  |  |  |  |
|  |  | XGB | 0.295 | <0.001 | ref |  |  |  |  |  |  |
|  | Model 2 | LR | <0.001 | <0.001 | <0.001 | ref |  |  |  |  |  |
|  |  | RF | <0.001 | <0.001 | <0.001 | <0.001 | ref |  |  |  |  |
|  |  | XGB | <0.001 | <0.001 | <0.001 | <0.001 | <0.001 | ref |  |  |  |
|  | Model 3 | LR | <0.001 | <0.001 | <0.001 | <0.001 | <0.001 | <0.001 | ref |  |  |
|  |  | RF | <0.001 | <0.001 | <0.001 | <0.001 | <0.001 | 0.004 | <0.001 | ref |  |
|  |  | XGB | <0.001 | <0.001 | <0.001 | <0.001 | <0.001 | <0.001 | <0.001 | <0.001 | ref |
| External validation | Model 1 | LR | ref |  |  |  |  |  |  |  |  |
|  |  | RF | <0.001 | ref |  |  |  |  |  |  |  |
|  |  | XGB | 0.63 | <0.001 | ref |  |  |  |  |  |  |
|  | Model 2 | LR | <0.001 | <0.001 | <0.001 | ref |  |  |  |  |  |
|  |  | RF | <0.001 | <0.001 | <0.001 | <0.001 | ref |  |  |  |  |
|  |  | XGB | <0.001 | <0.001 | <0.001 | 0.689 | <0.001 | ref |  |  |  |
|  | Model 3 | LR | <0.001 | <0.001 | <0.001 | 0.028 | <0.001 | 0.055 | ref |  |  |
|  |  | RF | <0.001 | <0.001 | <0.001 | 0.367 | <0.001 | 0.443 | 0.019 | ref |  |
|  |  | XGB | <0.001 | <0.001 | <0.001 | 0.130 | <0.001 | 0.038 | 0.709 | 0.01 | ref |

LR, logistic regression analysis; RF, random forest; XGB, extreme gradient boosting.

Supporting information figure 1. Calibration plots of Model 3 for internal and external validation datasets.


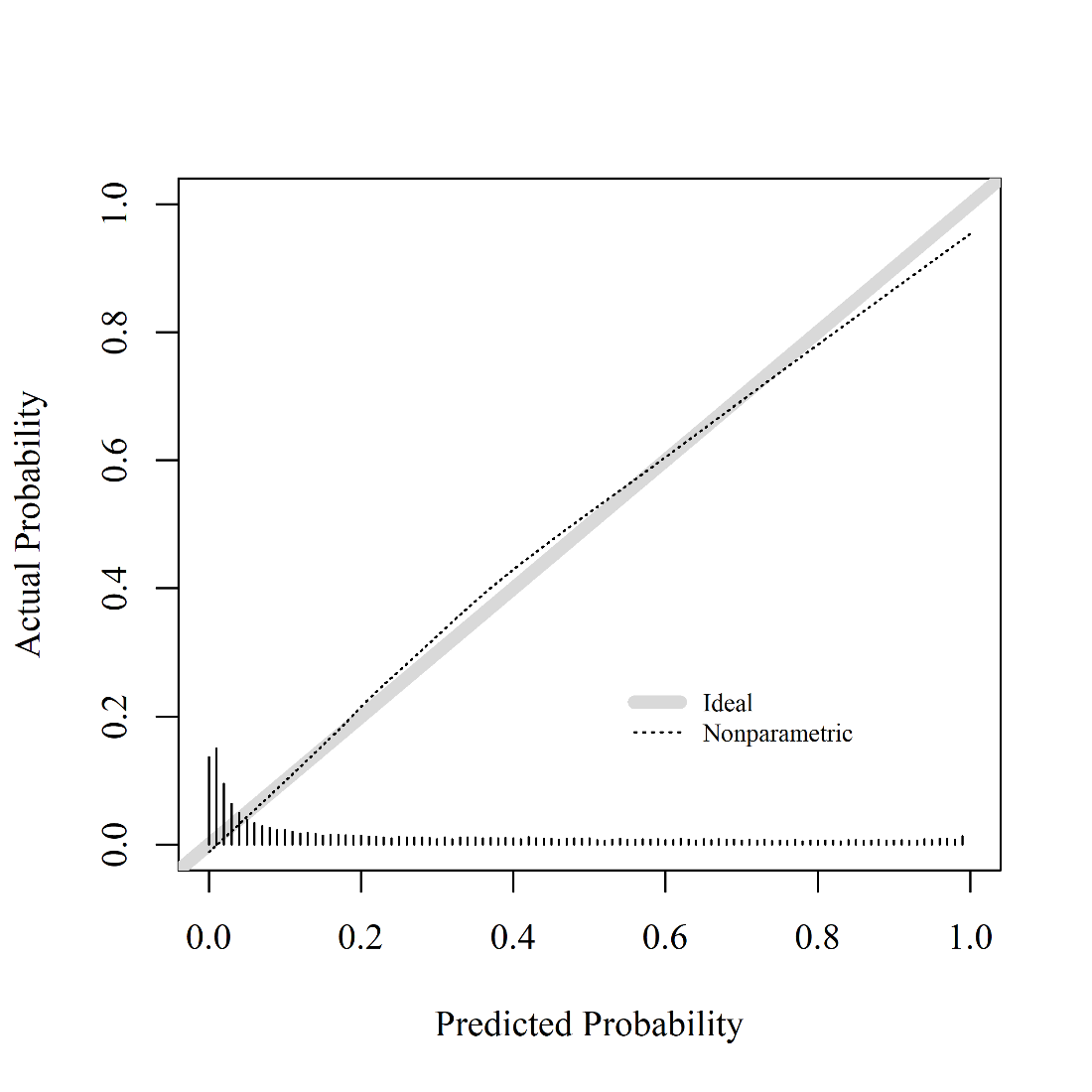


1. Calibration plot of Model 3 using logistic regression in the internal validation dataset


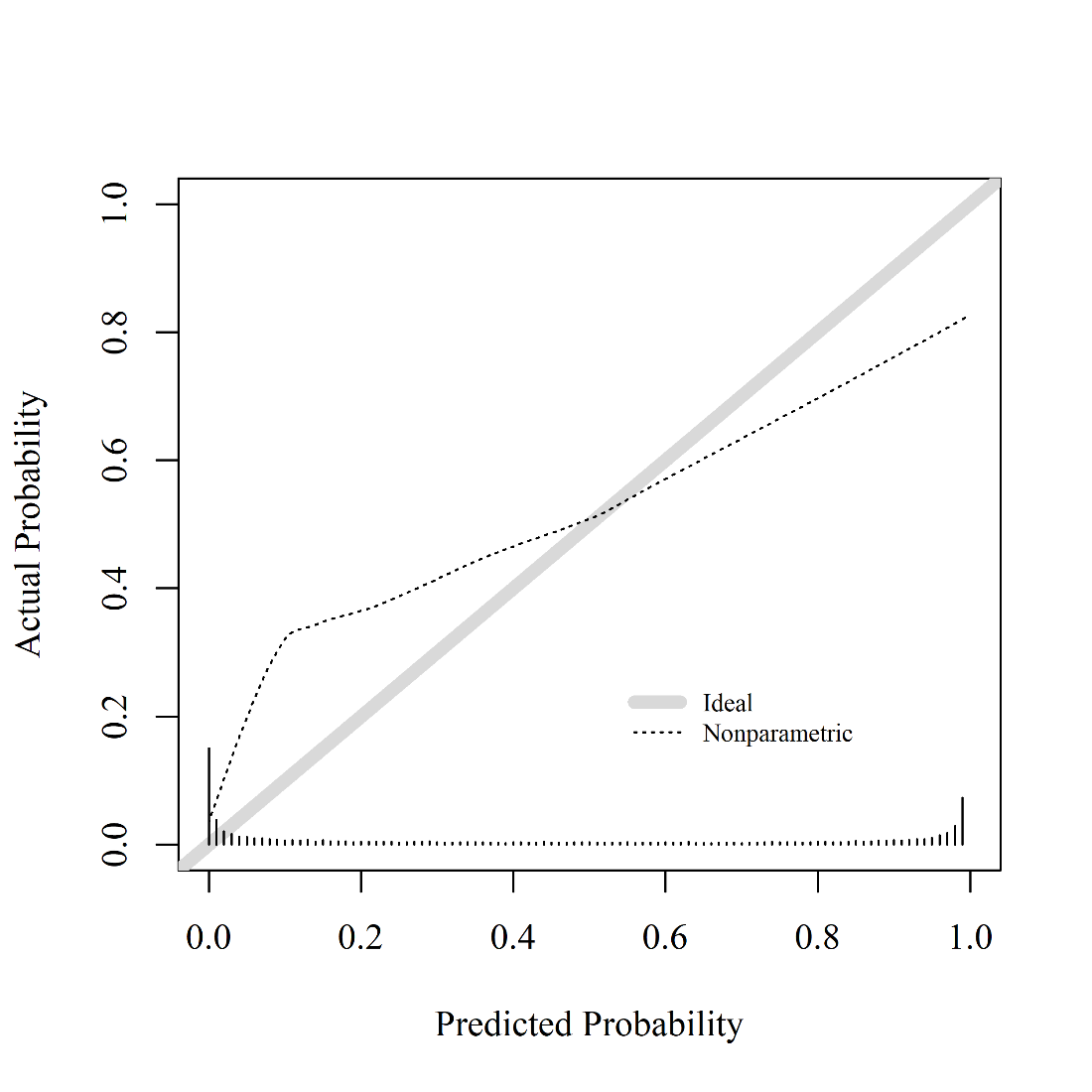


1. Calibration plot of Model 3 using random forest in the internal validation dataset


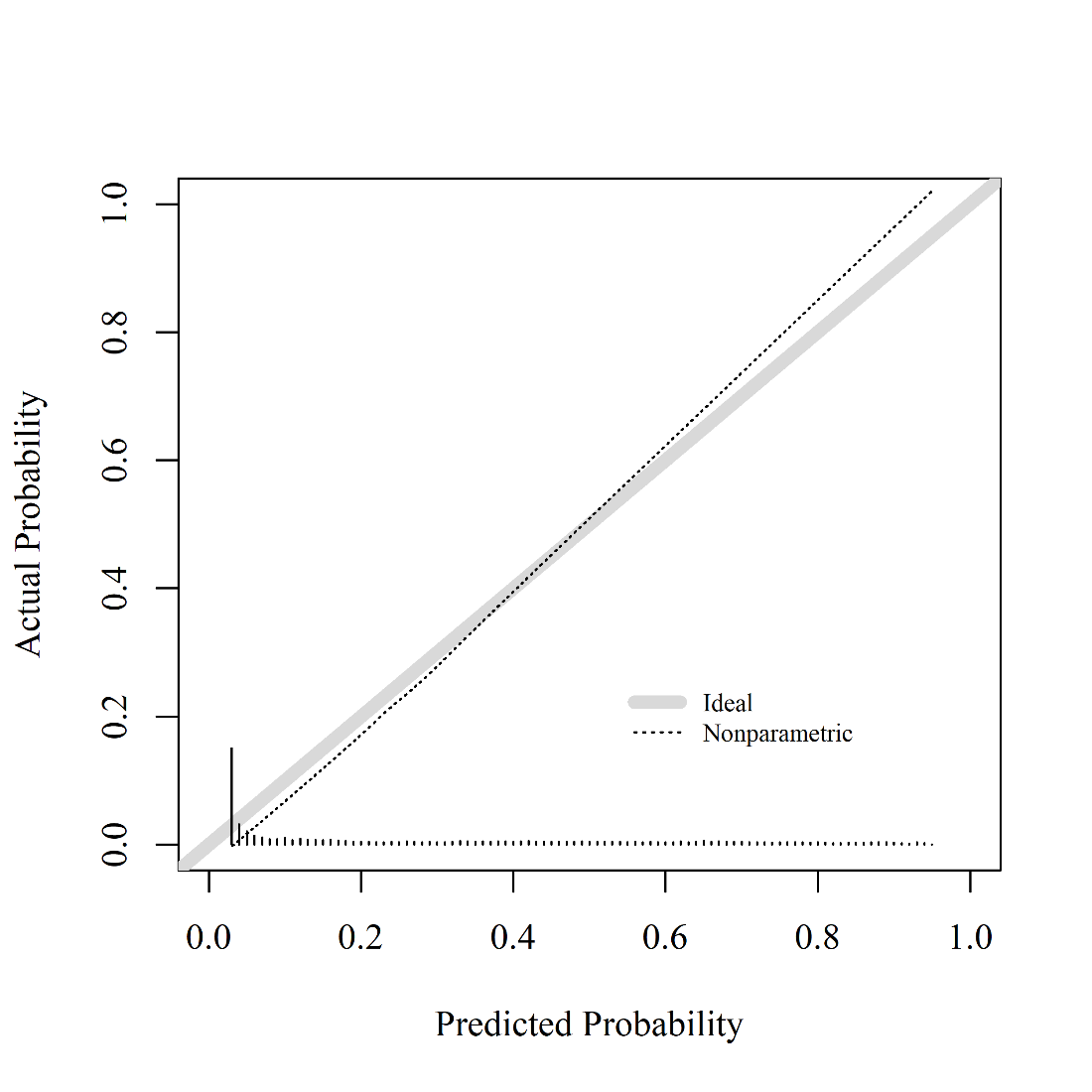


(C) Calibration plot of Model 3 using XGBoost in the internal validation dataset


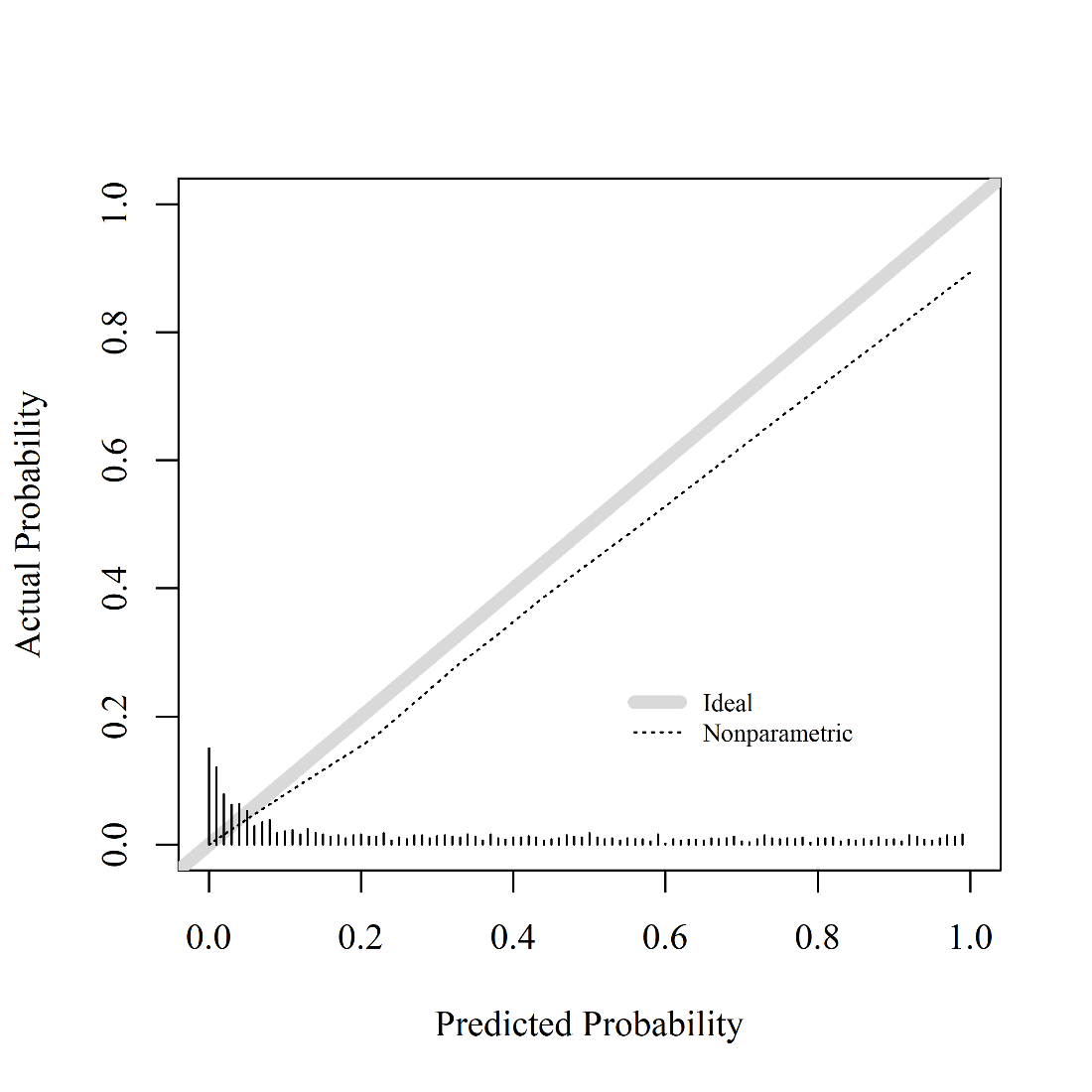


1. Calibration plot of Model 3 using logistic regression in the external validation dataset


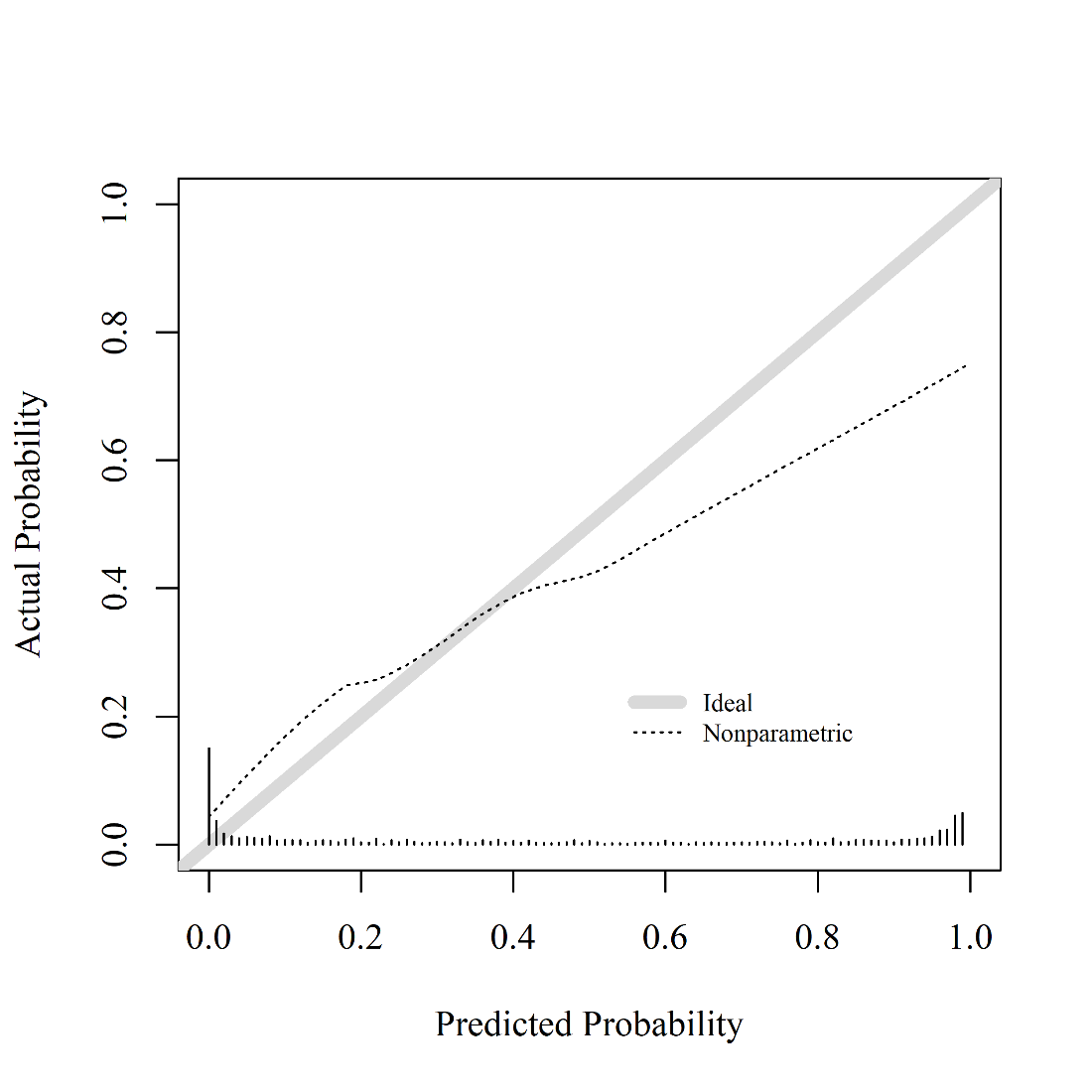


(E) Calibration plot of Model 3 using random forest in the external validation dataset


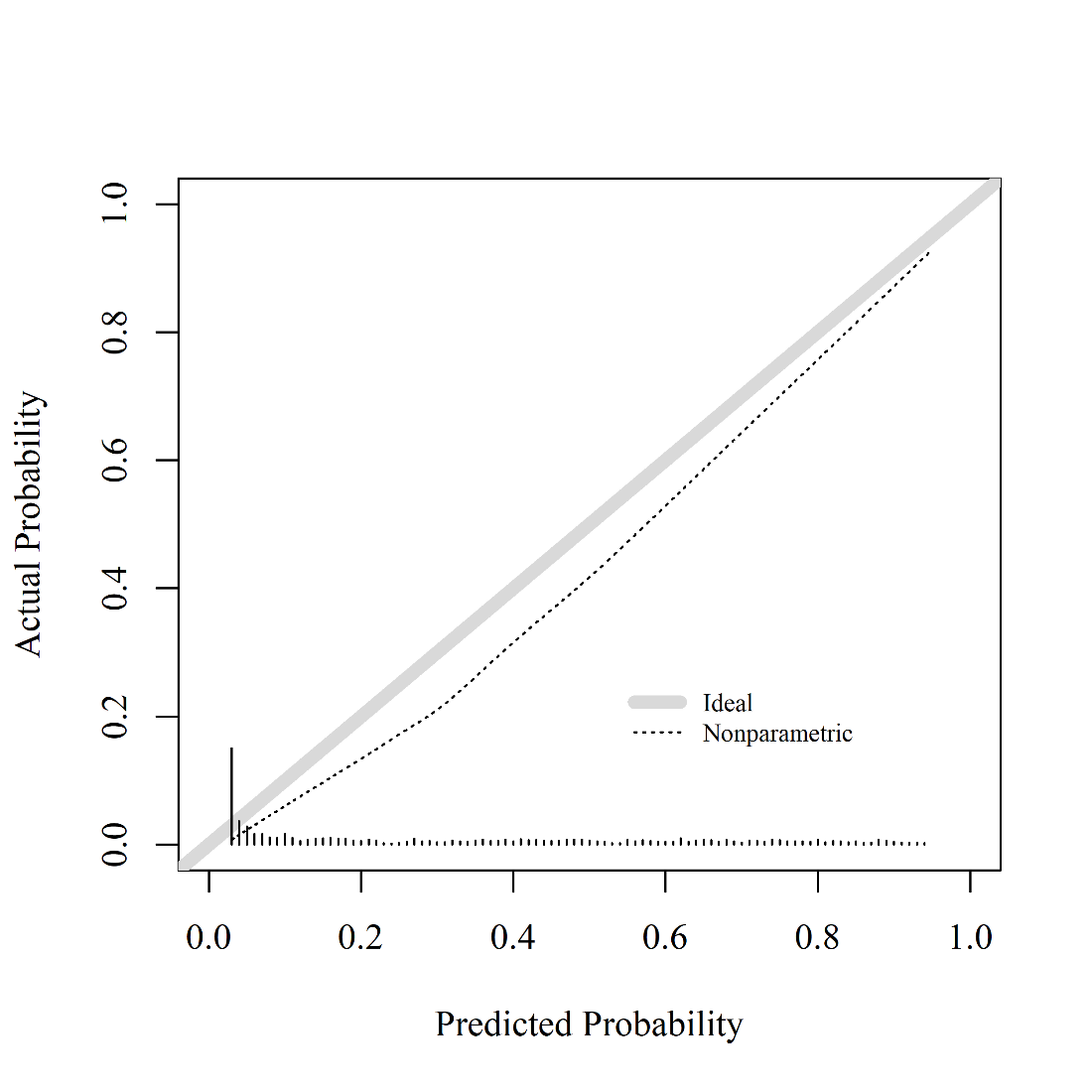


(F) Calibration plot of Model 3 using random forest in the external validation dataset

XGB, extreme gradient boosting.

Supporting information figure 2. ROC curves of PBF for the BMI-stratified logistic regression models in internal and external validation datasets.


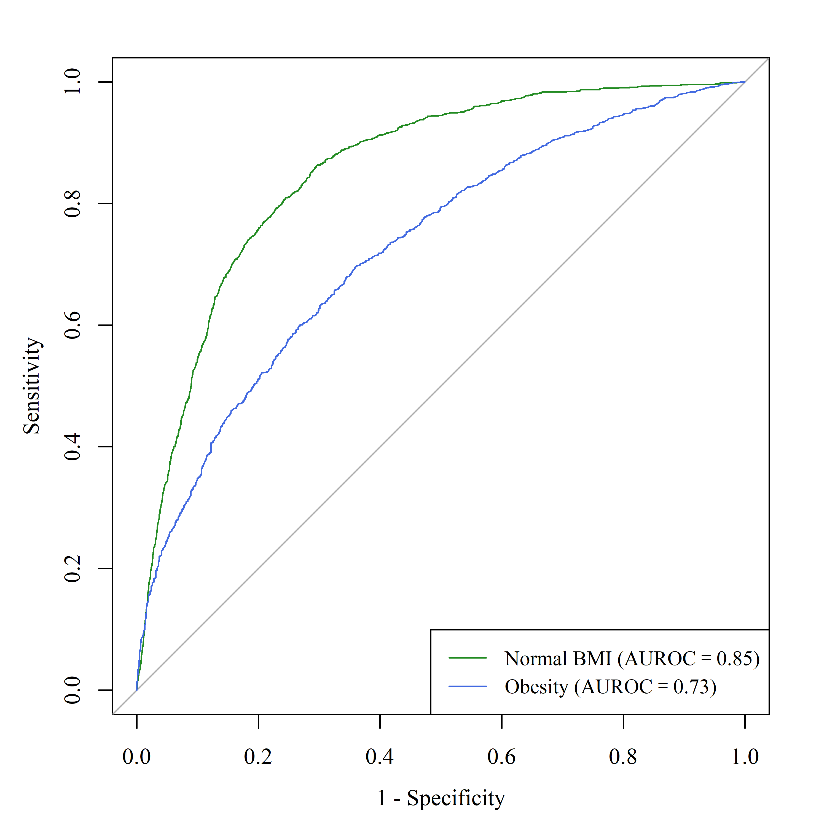


(A) ROC Curves of PBF for BMI-stratified Models (Internal Validation)


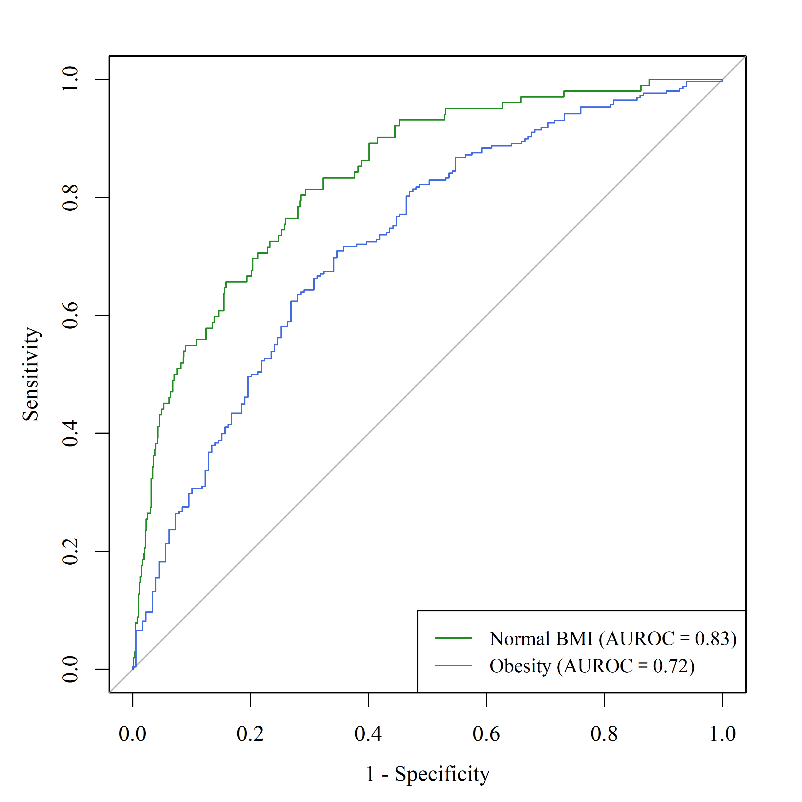


(B) ROC Curves of PBF for BMI-stratified Models (External Validation)

Supporting information figure 3. Nomogram with scoring system


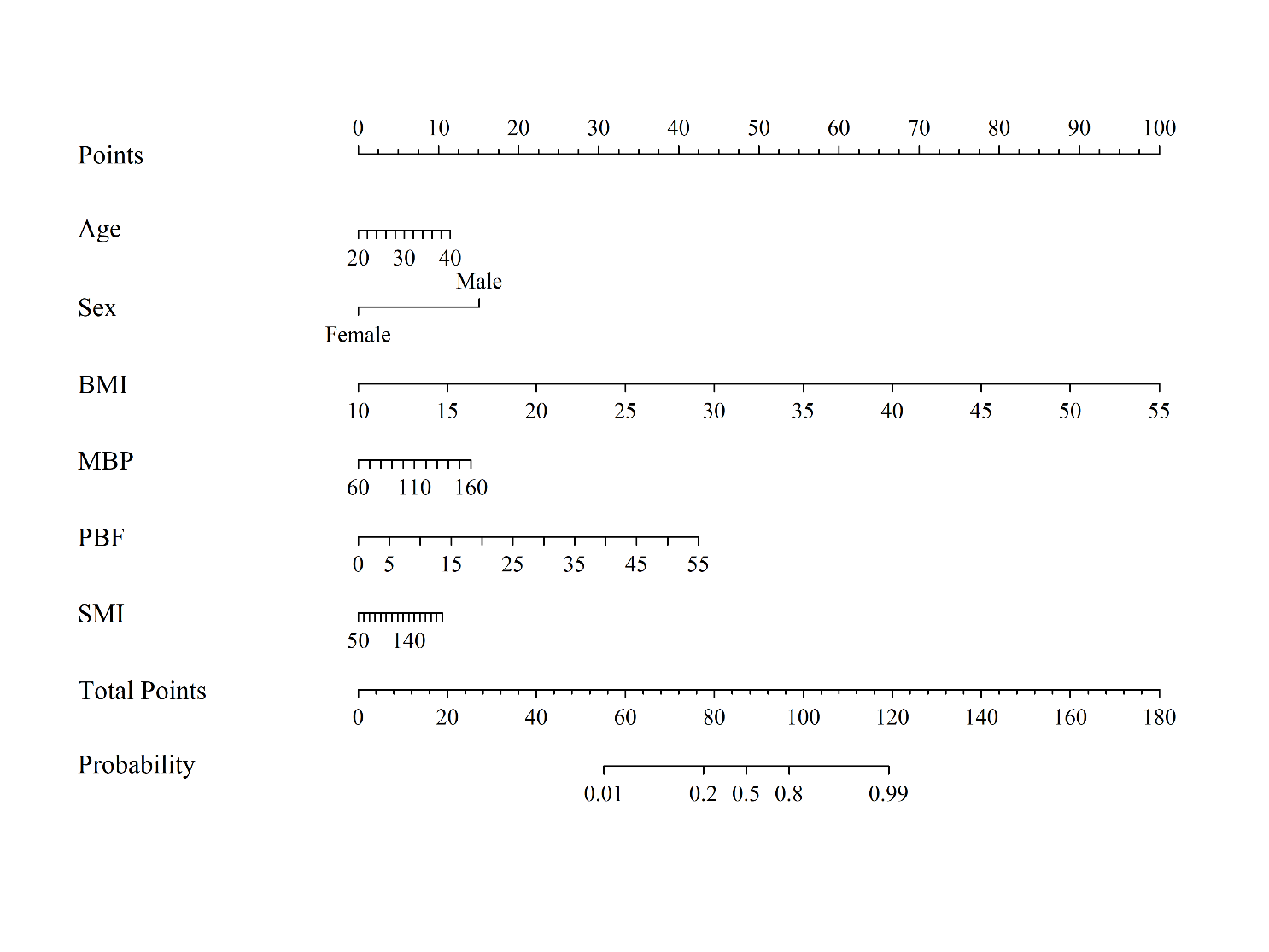


The scoring system calculates the probability of developing MASLD based on individual parameter contributions.

BMI, body mass index; MBP, mean blood pressure; PBF, percentage of body fat; SMI, skeletal muscle mass index.

Supporting information figure 4. SHAP summary plots of the logistic regression model according to age group in external validation


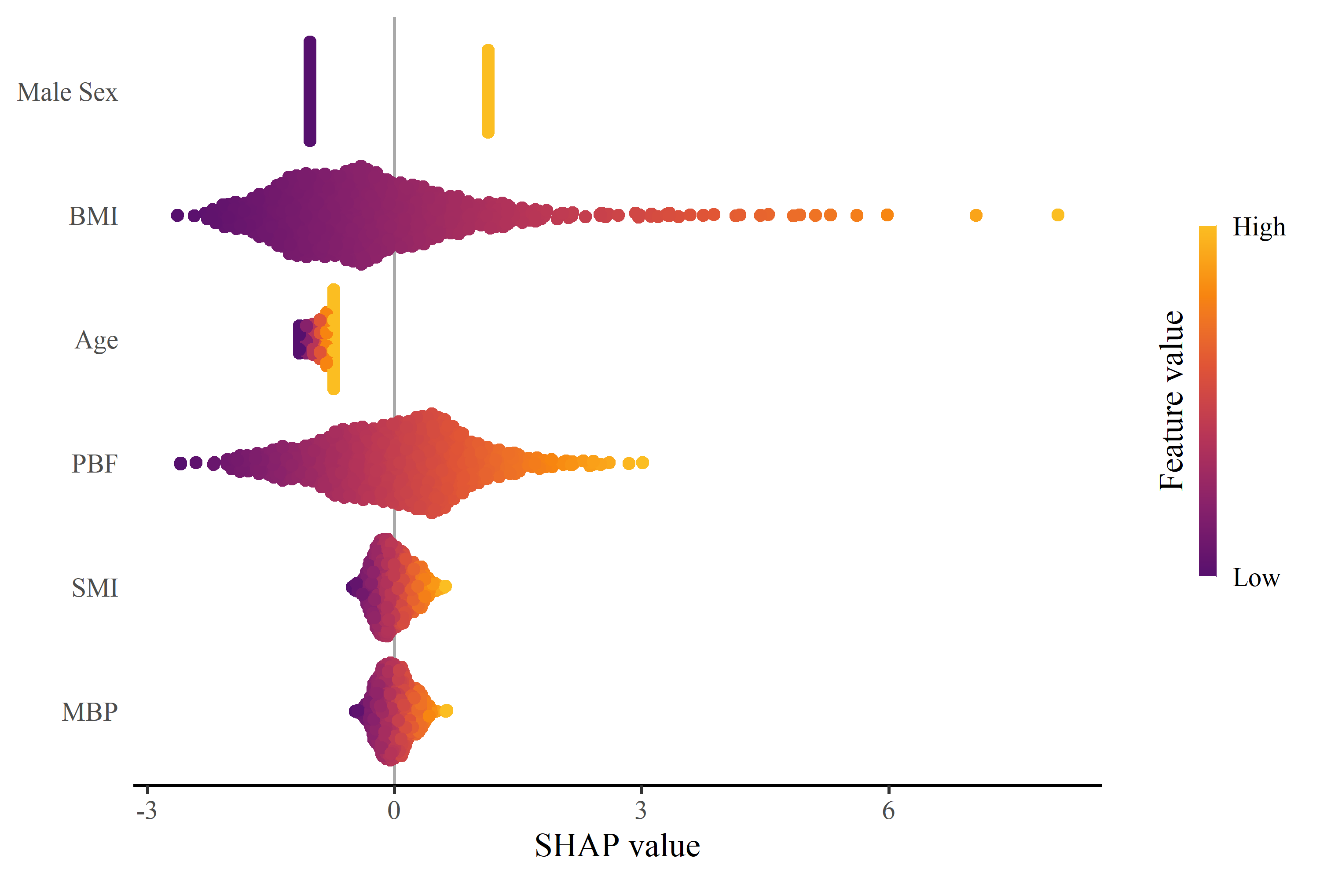


1. SHAP summary plot for participants aged 20–25 years


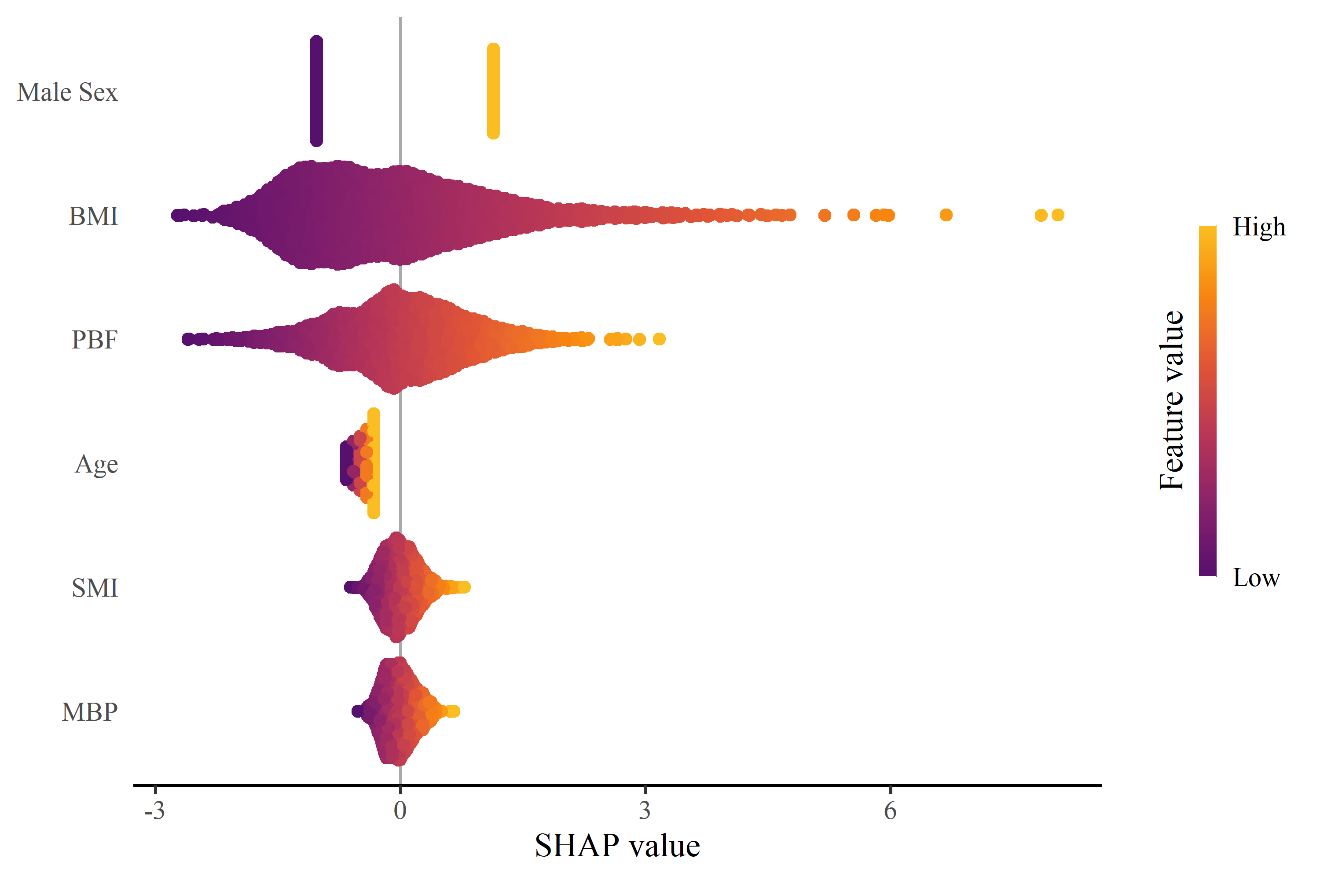


1. SHAP summary plot for participants aged 26–30 years


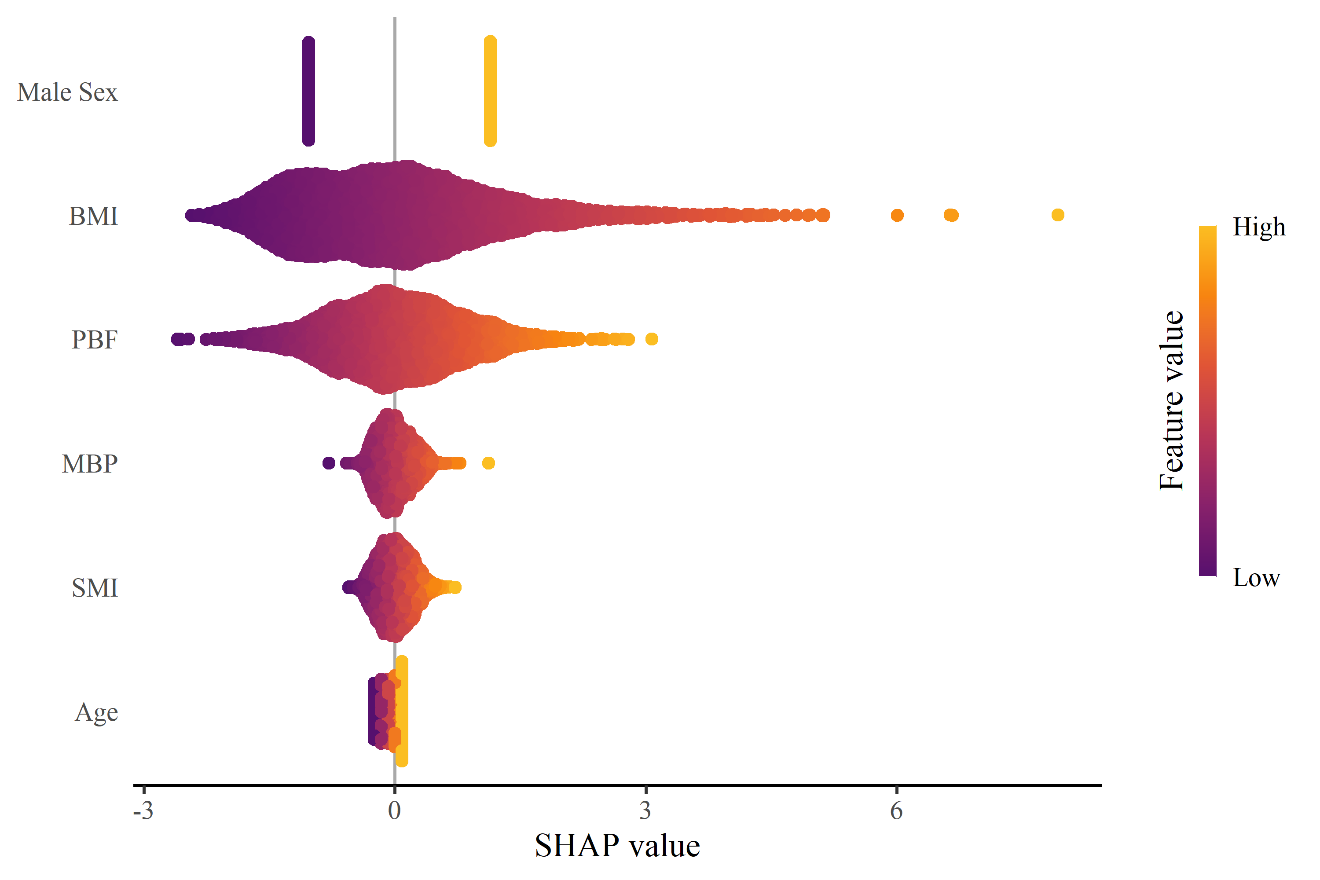


1. SHAP summary plot for participants aged 31–35 years


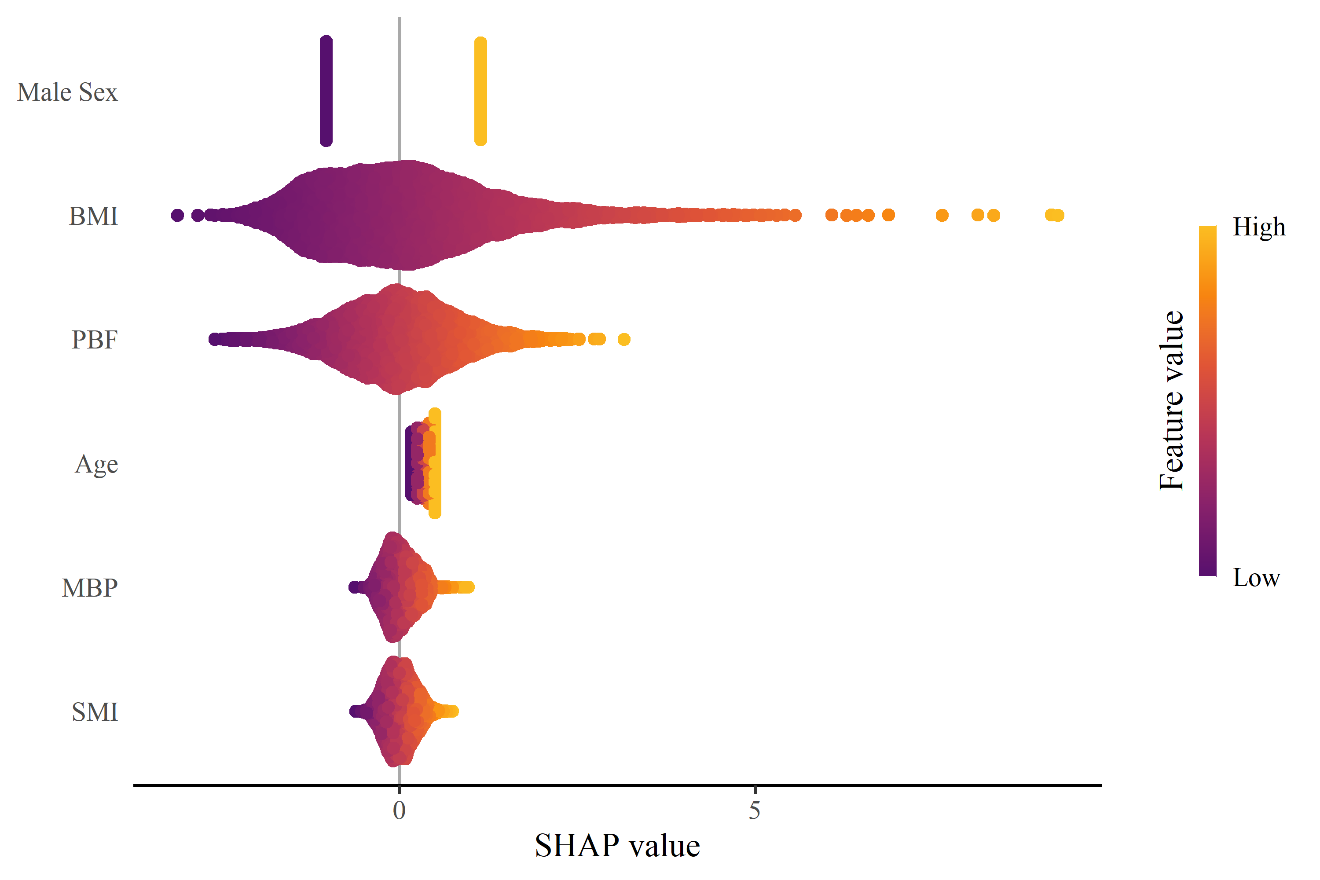


1. SHAP summary plot for participants aged 36–40 years

SHAP, Shapley additive explanation; MASLD, metabolic dysfunction-associated steatotic liver disease; BMI, body mass index; PBF, percentage of body fat; MBP, mean blood pressure; SMI, skeletal muscle mass index.

Supporting information figure 5. SHAP summary plots of the logistic regression model according to BMI category in external validation


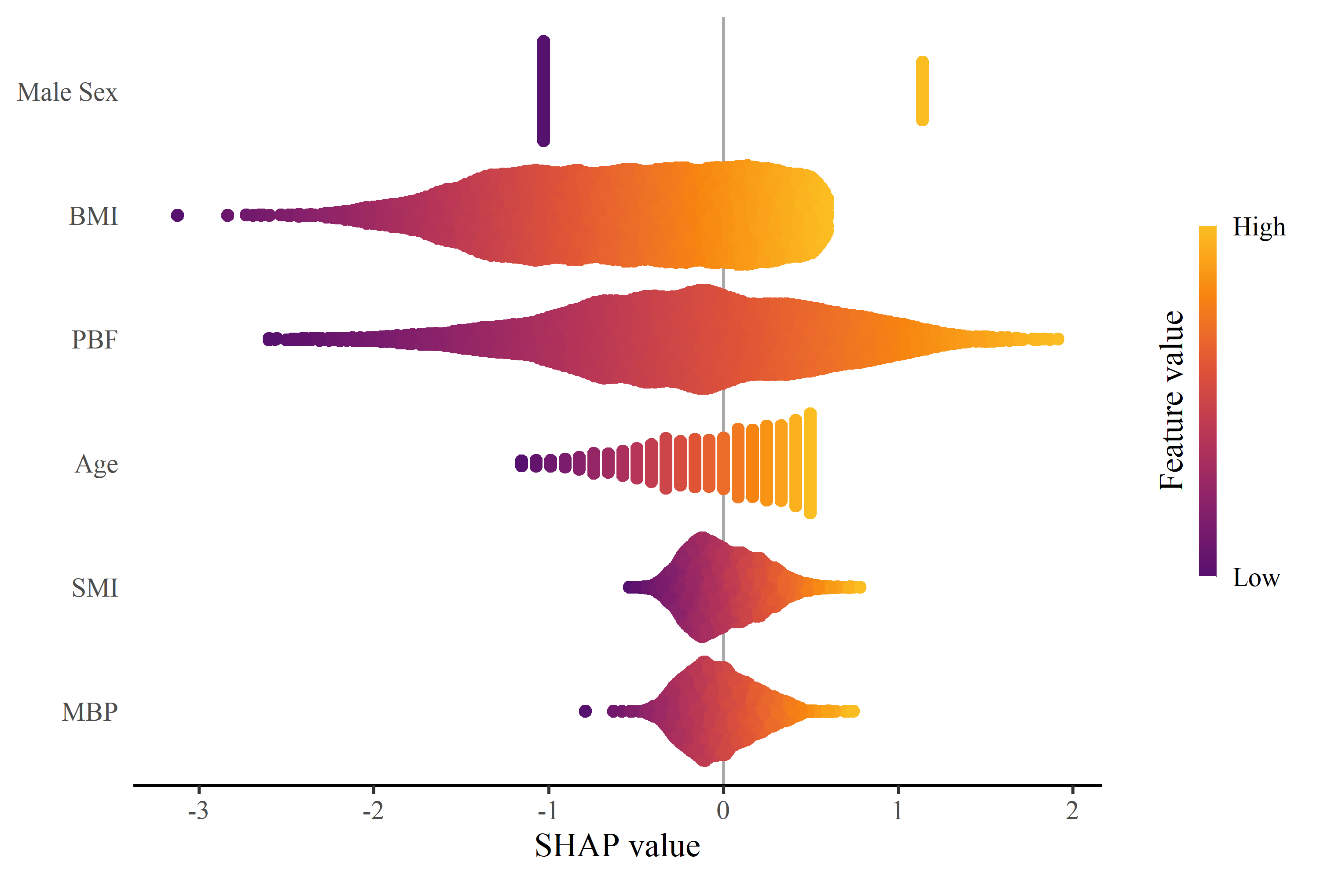


1. SHAP summary plot for the normal BMI group


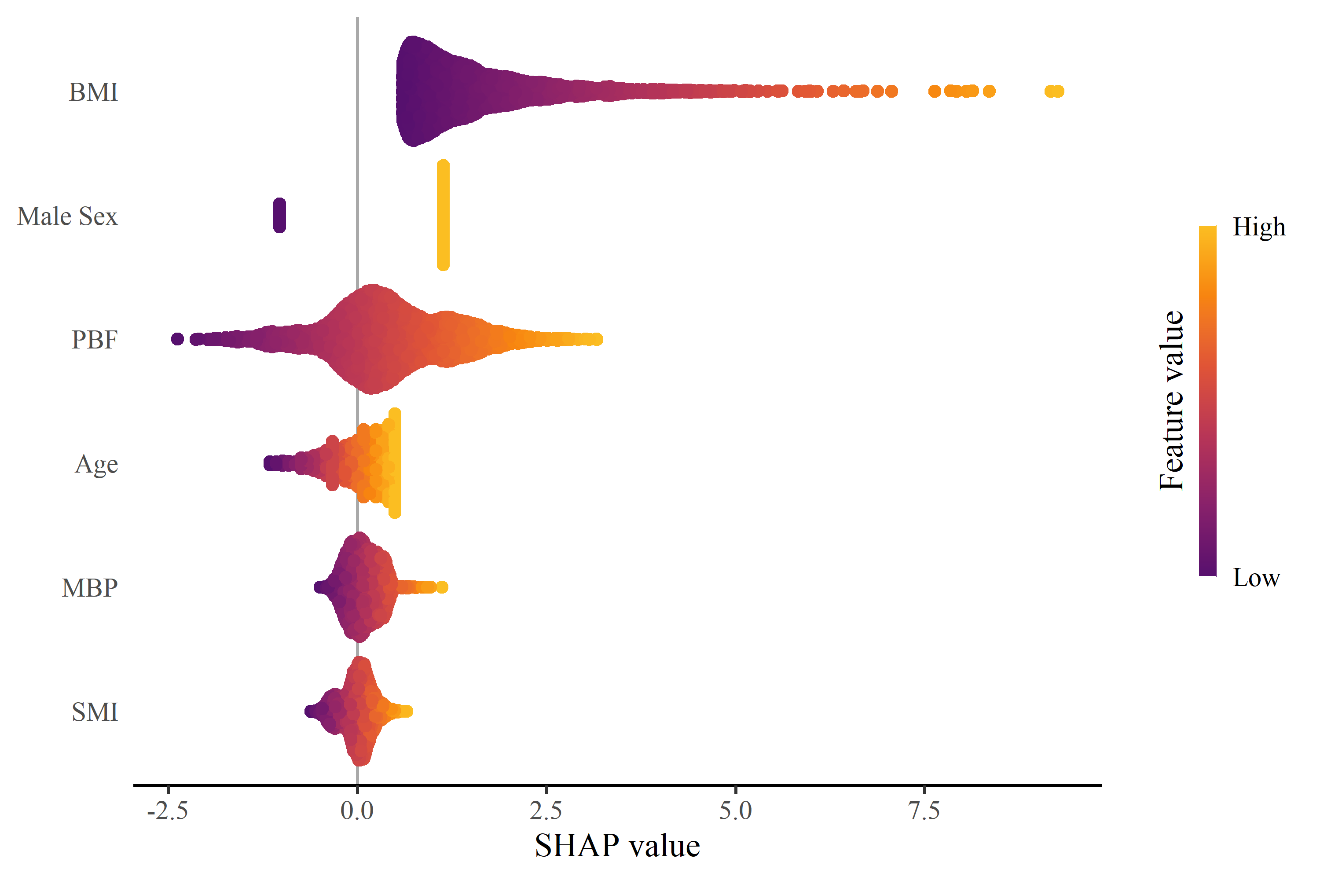


1. SHAP summary plot for the obesity group

SHAP, Shapley additive explanation; MASLD, metabolic dysfunction-associated steatotic liver disease; BMI, body mass index; PBF, percentage of body fat; MBP, mean blood pressure; SMI, skeletal muscle mass index.
